# Thin Films of BaM Hexaferrite with an Inclined Orientation of the Easy Magnetization Axis: Crystal Structure and Magnetic Properties

**DOI:** 10.3390/nano14231883

**Published:** 2024-11-23

**Authors:** Boris Krichevtsov, Alexander Korovin, Vladimir Fedorov, Sergey Suturin, Aleksandr A. Levin, Andrey Telegin, Elena Balashova, Nikolai Sokolov

**Affiliations:** 1 Ioffe Institute, Politechnicheskaya 26, 194021 St. Petersburg, Russia; amkorovin@mail.ioffe.ru (A.K.); suturin@mail.ioffe.ru (S.S.); aleksandr.a.levin@mail.ioffe.ru (A.A.L.); balashova@mail.ioffe.ru (E.B.); nsokolov@fl.ioffe.ru (N.S.); 2 Laboratory of Renewable Energy Sources, Alferov University, Khlopin St. 8/3, 194021 St. Petersburg, Russia; burunduk.uk@gmail.com; 3 M.N. Mikheev Institute of Metal Physics, 18 S. Kovalevskaya Str., 620108 Yekaterinburg, Russia; telegin@imp.uran.ru

**Keywords:** hexaferrite, molecular beam epitaxy, crystal structure, magnetic anisotropy, hysteresis loops, XRD, magneto-optical Kerr effect, VSM, AFM

## Abstract

Thin (~50 nm thick) BaM hexaferrite (BaFe_12_O_19_) films were grown on (1–102) and (0001) cut α-Al_2_O_3_ (sapphire) substrates via laser molecular beam epitaxy using a one- or two-stage growth protocol. The advantages of a two-stage protocol are shown. The surface morphology, structural and magnetic properties of films were studied using atomic force microscopy, reflected high-energy electron diffraction, three-dimensional X-ray diffraction reciprocal space mapping, powder X-ray diffraction, magneto-optical, and magnetometric methods. Annealed BaFe_12_O_19_/Al_2_O_3_ (1–102) structures consist of close-packed islands epitaxially bonded to the substrate. The hexagonal crystallographic axis and the easy axis (EA) of the magnetization of the films are deflected from the normal to the film by an angle of *φ*~60°. The films exhibit magnetic hysteresis loops for both in-plane **H**_in-plane_ and out-of-plane **H**_out-of-plane_ magnetic fields. The shape of *M*_out-of-plane_(*H*_in-plane_) and *M*_in-plane_(*H*_in-plane_) hysteresis loops strongly depends on the azimuth *θ* of the **H**_in plane_, confirming the tilted orientation of the EA. The *M*_out-of-plane_(*H*_out-of-plane_) magnetization curves are caused by the reversible rotation of magnetization and irreversible magnetization jumps associated with the appearance and motion of domain walls. In the absence of a magnetic field, the magnetization is oriented at an angle close to *φ*.

## 1. Introduction

Limitations in the speed and volume of the transmitted information in modern computer chips, the operation of which is based on the use of electric currents, are associated with the undesirable release of Joule heat. Joule heat causes very strong heating of the processor and necessitates the use of cooling devices, which also require electrical energy for their operation. In this regard, it is worth noting that, at present, the information, communication, and technology sectors using computers consume from 5% to 9% of the electricity produced in the world, and by 2030, this figure is expected to increase to 20% [1], since the amount of energy used for computing is growing exponentially. Therefore, reducing the energy for heating and eliminating cooling elements can lead to significant savings in electricity.

As shown in a number of works, the problem of Joule heat can be solved by using spin wave (SW) packets for transmitting and processing information via monolithic microwave integrated circuits [2,3,4,5]. To excite, control, and receive SWs, thin-film magnetic materials with low SW losses are required, as well as the ability to control their direction, which is possible with an out-of-plane magnetization orientation, and operating at frequencies of *f*~50–100 GHz. For these reasons, different magnetic materials have been used to fabricate thin-film magnetic structures applying various methods.

Particular attention has been paid to yttrium iron garnet (YIG) heterostructures, since bulk YIG single crystals exhibit very low ferromagnetic resonance (FMR) linewidths, indicating extremely narrow SW damping [5]. However, due to the relatively small values of the magnetic anisotropy field, YIG thin films grown on gadolinium gallium garnet (GGG) or neodymium gallium garnet (NdGG) exhibit an in-plane magnetization orientation, and a significant magnetic field of ~2 kOe is required to implement out-of-plane orientation [6,7,8]. Also, the relatively low saturation magnetization (4*πM*_S_ = 1790 G at room temperature) limits the frequency range for microwave applications to ~10 GHz.

Thin films of ferrite spinels, in particular Ni ferrite, also attract much attention due to their promising magnetic properties [9,10,11,12,13]. As compared to YIG, Ni-ferrite exhibits higher values of magnetic anisotropy *H*_a_~340 Oe and saturation magnetization 4*πM*_S_~3.4 kG, which expands the frequency range for developing superhigh frequency (SHF) phase shifters, filters, etc. However, an out-of-plane magnetization has not yet been observed in these films at room temperature.

Promising materials for the above mentioned purposes are hexaferrites with a uniaxial crystal structure of hexagonal syngony (space group *P*6_3_/*mmc* (194); see, for example, Ref. [14]) and magnetic anisotropy [15,16,17,18,19,20,21,22]. In particular, Ba-hexaferite of the M type (BaM) with the chemical formula BaFe_12_O_19_ has high values of the uniaxial anisotropy field *H*_a_~18 kOe, that can be controlled via the partial substitution of Ba and Fe ions, and saturation magnetization 4*πM*_s_~4.6 kG, which makes it possible to implement devices operating at frequencies of up to 50–100 GHz. The presence of strong uniaxial magnetic anisotropy enables the obtaining of thin films of BaM hexaferrite, in which, in the absence of a magnetic field *H*, a single-domain state with a magnetization orientation normal to the plane of the structure is realized [15,16].

Though the magnetic energy of the hexaferrites is an order lower than that of rare-earth-based magnets [23], hexaferrites have such advantages as easy moldability, light weight, and low cost. In addition, hexaferrite crystals and films can have a relatively narrow FMR line. The possibility of realizing a single-domain state in hexaferrite crystals and films without applying a magnetic field makes them very attractive for developing planar microwave devices. Moreover, the ability to change the orientation of the magnetization with a relatively small magnetic field in hexaferrites allows the changing of the frequency of spin waves over a wide range.

Films of BaM hexaferrite have been prepared by using different methods (liquid phase epitaxy, magnetron sputtering, pulsed laser deposition, screen printing, laser molecular beam epitaxy (LMBE), and metal–organic decomposition) on various substrates (sapphire, MgO, GGG, and 6H–SiC). The results of the studies of film morphology, crystal structure, and magnetic properties have been summarized in the review articles [15,16,17]. It is shown that in order to create films that simultaneously exhibit a small FMR linewidth and a high value of out-of-plane remnant magnetization *M*_rem_ (self-bias), their crystal structure must include a certain number of defects that can stabilize *M*_rem_ without a significant increase in SW attenuation. In films with perfect crystal structures, *M*_rem_ is close to zero due to the formation of a domain structure at *H* = 0 (self-bias paradox [16]).

In [24], thin films (thickness *h*~50 nm) of BaFe_12_O_19_ were fabricated on Al_2_O_3_ (0001) substrates (α-Al_2_O_3_ or simply Al_2_O_3_ in the text; trigonal space group $R\bar{3}c$ (167) in a hexagonal setting, for example, Ref. [25]) using the LMBE method. After post-growth annealing in air at *T*_ann_ = 1000 °C, they reveal almost rectangular hysteresis loops for the out-of-plane magnetic field *H* with the ratio *M*_rem_/*M*_s_ (self-bias) close to 100%. Increasing the film thickness up to *h*~300–500 nm does not lead to a significant decrease in self-bias, but the hysteresis loops become less rectangular. This indicates that an increase in thickness is accompanied by an increase in crystalline and magnetic inhomogeneities, leading to inclined wings of the hysteresis loops. On the other hand, such inhomogeneities may also present in 50 nm thick films and provide remarkable self-bias. One of the goals of this work was to show that a decrease in film uniformity with a thickness increase can be compensated by a multi-stage growth protocol.

It should be noted that the structures with an inclined orientation of remnant magnetization *M*_rem_ are also attractive for the applications. In this case, the switching of the magnetization or control of the magnetization orientation is possible both via a magnetic field oriented in the plane of the film (*H*_in-plane_) and perpendicular to the plane (*H*_out-of-plane_). A comparison of BaM films obtained via reactive magnetron sputtering on sapphire substrates of A—(11–20), R—(1–102), and C—(0001) cuts, carried out in [26], suggested that the mutual orientations of the crystal structure of BaM film and the sapphire substrates in epitaxial structures are the same for different substrate orientations. As a result, thin films of BaM hexaferrite film with an easy axis of magnetization (EA) at any angle with respect to the substrate can be obtained by making an appropriate choice of the orientation of the sapphire substrate. The in-plane orientation of the BaM *c*-axis has been realized on M-planes (1–100) of Al_2_O_3_ by using the liquid phase epitaxy technique [27]. Epitaxial BaM hexaferrite films of different thicknesses with an in-plane oriented *c*-axis were grown on an A-plane (11–20) by using direct current (DC) magnetron sputtering followed by an ex situ annealing process [28]. A conical magnetic structure, which is required to establish the magnetoelectric effect in hexaferrites, has been observed in BaFe_10.2_Sc_1.8_O_19_ films grown on Al_2_O_3_ (0001) substrates via pulsed laser deposition [29].

Taking into account the results of previous works [24,26,27,28,29], it was expected that in epitaxial films grown using LMBE on R-cut (1–102) sapphire substrates, the orientation of the hexagonal axis and, accordingly, the EA will be deviated from the normal to the substrate surface by a significant angle, and in the absence of a magnetic field, the tilted orientation of the magnetization will be preserved throughout the volume of the film. In this case, it becomes possible to switch the magnetization, by applying both out-of-plane and in-plane magnetic fields and effectively controlling the magnitude of the magnetization component normal to the plane of the film. This can enable the tuning of the frequency of spin waves over a wide range. In addition, the state with an inclined orientation of the magnetization can be useful for conducting experiments on the magnetoelectric effect in the films.

In this work, we report the fabrication of thin epitaxial BaM hexaferrite films on Al_2_O_3_ (1–102) (R-cut) substrate by using the LMBE method, as well as the study of their surface morphology, crystal structure, and static magnetic properties. For convenience, we will use the designation (1–102) for the R-cut of α-Al_2_O_3_ instead of the equivalent (01–12) or (10–12). In addition, the results of the fabrication of BaFe_12_O_19_/Al_2_O_3_ (0001) heterostructures using a two-stage growth protocol and the study of their magnetic properties are presented. A comparison of the results obtained using different growth protocols was intended to answer the following questions:

(1) Does film growth on an already formed crystal structure of BaM hexaferrite lead to the formation of this structure throughout the volume without additional annealing?
(2) How does the value of film thicknesses affect the magnetic properties of the structure obtained using the two-step protocol?
(3) Is it possible to increase the film thickness whilst keeping the rectangular shape of the hysteresis loops?

Thus, the main objectives of the work were as follows:

- to grow by using the LMBE method epitaxial BaM films on the anisotropic (1–102) surface of Al_2_O_3_;
- to check whether the expected crystalline anisotropy of the film will result in an incline from the surface normal direction of the remanent magnetization which is attractive for a number of applications;
- to explore possibility of application of two- or multistage growth protocol to improve crystalline and magnetic properties of thicker BaM hexaferrite films.

## 2. Materials and Methods

BaM hexaferrite (BaFe_12_O_19_) layers with a thickness *h* = 20–170 nm were grown on Al_2_O_3_ (0001) (C-cut) and Al_2_O_3_ (1–102) (R-cut) sapphire substrates via the LMBE technique in a setup designed for the growth of oxide layers (Surface GmbH, Hückelhoven, Germany). The growth of the structures was carried out in an oxygen atmosphere at a pressure *p* of 0.05–0.06 mbar and growth temperature *T*_gr_ = 700 °C. Commercial leucosapphire α-Al_2_O_3_ (0001) and α-Al_2_O_3_ (1–102) wafers with the thickness of 0.5 mm and average root mean square (RMS) surface roughness *RMS* < 2 nm were used as substrates. The growth of BaM hexaferrite films was carried out via the ablation of a stoichiometric BaFe_12_O_19_ target with short (*τ* = 10 ns) pulses from an excimer KrF laser (wavelength *λ* = 248 nm) with an energy density of ~2 J/cm^2^. For the manufacture of the target, a standard technology for the preparation of ferrites from a charge consisting of BaCO_3_ and Fe_2_O_3_ oxides of a reagent degree of purity was used. To carry out post-growth annealing, the grown structure was removed after cooling from the growth chamber and placed in a muffle furnace, in which it was heated to a temperature of *T_ann_ =* 1000 °C and annealed in air for *τ_ann_* = 30 or 60 min (see Table 1). A Jipelec JetFirst 100 (SemiStar Corp., Morgan Hill, CA, USA) rapid thermal annealing system was used for the annealing in a nitrogen atmosphere.

The growth rate was calibrated using a quartz monitor. The thickness of the grown films was calculated as the product of the growth rate and growth time. The estimated standard deviations (e.s.d.s.) in the thickness measurements are given in Table 1.

The growth of the structures was carried out using either a one-stage (BaFe_12_O_19_/Al_2_O_3_ (1–102), sample #1, and BaFe_12_O_19_/Al_2_O_3_ (0001), samples #2, #4, and #6) or two-stage (BaFe_12_O_19_/Al_2_O_3_ (0001), samples #3 and #5) growth protocol. The one-stage protocol included the growth process, removing the sample from the growth chamber, and annealing in air at *T*_ann_ = 1000 °C (excluding sample #6, which was annealed in the growth chamber at an oxygen pressure of *p* = 0.5 mBar). When using a two-stage protocol, the growth of a thick layer is divided into growth-annealing cycles, and in each of them, the growth of a layer at a temperature of *T*_gr_ = 700 °C alternates with its annealing in air at a temperature of *T*_ann_ = 1000 °C. Accordingly, a hexaferrite layer 20 nm or 50 nm thick was first grown on the substrate at *T*_gr_ = 700 °C (samples #2 and #4, respectively). Then, the resulting heterostructure was annealed in a muffle furnace in an air atmosphere at *T*_ann_ = 1000 °C during a time *τ*_ann_ (from 10 min to one hour). In the second stage, the structure was again loaded into the growth chamber, and a second layer 150 nm or 50 nm thick was grown on it using the same technique (structures #3 and #5, respectively). The magnetic hysteresis loops were measured at each stage both before and after the annealing process. The substrates used, as well as the growth and annealing parameters of the studied samples, are presented in Table 1.

As can be seen in Table 1, samples #1 and #6 were prepared in two growth experiments. This was carried out to test the reproducibility of the structural and magnetic properties. Further in the text, the experimental data are given only for one of the samples (they are similar for the other). In this work, only BaM hexaferrite films prepared on an Al_2_O_3_ (1–102) substrate (sample #1) were studied by using the powder X-ray diffraction (XRD) method. The results of the XRD study of BaM hexaferrite layers prepared on an Al_2_O_3_ (0001) substrate with similar growth parameters are given in [24].

Examples of the photos of the prepared film-samples are presented in Section S1 of the Supplementary Materials (Figures S1–S3).

The structural and magnetic properties of the grown and annealed films were investigated by means of atomic force microscopy (AFM), mapping of the reflection high electron diffraction (RHEED), XRD three-dimensional (3D) reciprocal space mapping, powder XRD, vibrating sample magnetometry (VSM), and the magneto-optical Kerr effect (PMOKE). All these techniques are described in detail in [24]. Below, we will give only a brief description of the methods used.

The surface morphology of the Al_2_O_3_ substrates and the films grown on them were studied using an Integra NT-MDT (Zelenograd, Russia) atomic force microscope in the semi-contact mode at room temperature. In addition, the surface morphology of the annealed BaFe_12_O_19_ layers was studied.

RHEED, measured using an attachment (Surface GmbH, Hückelhoven, Germany) installed in the growth chamber, was applied to monitor, in situ, the growth of the BaM hexaferrite films on the substrate, studying the crystal structure of the grown layers. During the RHEED measurements, the sample was rotated around the normal to the surface at an angle φ with a step Δφ = 0.5°, taking a series of the RHEED patterns at every step Δφ and constructing the 3D map of the diffraction intensity distribution in the reciprocal space and projections of the map diffraction intensity.

An ex situ technique of the XRD 3D reciprocal space mapping was applied to characterize the structure of the films after deposition and annealing. During the XRD measurements, the sample was rotated around the normal to the surface at an angle *φ* with a step Δ*φ* = 0.5°. Using a Super Nova diffractometer (Agilent Technologies, Inc., Santa Clara, CF, USA) (kappa geometry, a two-dimensional (2D) semiconductor detector (Atlas S2 CCD), and an X-ray gun with a copper cathode (*λ* = 1.5418 Å)), a series of XRD patterns was registered at every step Δ*φ*, when the samples were rotated around the normal to the sample surface. Using the original firm software, the 3D maps were assembled.

XRD measurements of the BaM hexaferrite films prepared on an Al_2_O_3_ (1–102) substrate (sample #1) were also carried out using a D2 Phaser powder diffractometer (Bruker AXS, Karlsruhe, Germany) with a linear semiconductor position-sensitive detector LYNXEYE (Bruker AXS, Karlsruhe, Germany) and an X-ray tube with a copper anode. Scanning in the symmetrical 2*θ*-*θ* mode in the range of diffraction angle 2*θ* = 6–140° with a step Δ2*θ*_step_ = 0.02° was carried out in vertical Bragg-Brentano *θ*-*θ* geometry using Cu-*K*_α_ radiation (*λ* = 1.5418 Å), filtered with a nickel-foil filter. To reduce the possible effects of preferential orientation, the samples during the XRD measurements were rotated around the axis of the sample holder, which coincided with the axis of the X-ray goniometer. Due to the peculiarities of the desktop X-ray diffractometer used, the temperature in the sample chamber during the measurements was 313 ± 1 K.

The X-ray phase analysis of the registered XRD patterns was performed using program EVA [30] and the Powder Diffraction File-2 (PDF-2) database, released in 2014 [31].

The observed Bragg angles (2*θ*_B_) of the reflections were corrected for angular corrections (zero shift Δ2θ_zero_ and displacement Δ2θ_displ_∙cos(θ)) (see [24] for details), obtained as a result of the additional measurements of the samples embedded in the powder XRD standard Si640f (NIST, Gaithersburg, MD, USA) so that the plane of the samples, which looked like plates, was flush with the plane of the powder standard, and both surfaces fell within the area illuminated by the X-ray beam. According to the value of the criteria *FWHM*/*B*_int_ = 0.85 (10) [32] averaged over all the observed reflections (where *FWHM* is the full width at half maximum and *B*_int_ is the integral width of the reflections, both without a correction for instrumental broadening), the XRD reflections were attributed to the reflections of pseudo-Voigt (pV) profile type. The instrumental contribution to the *FWHM* values was corrected using the procedure for pV reflections [33] resulting in the corrected value, *FWHM*_corr_. The mean crystallite size *D*_0_ in the model without microstrain was estimated by averaging the individual crystallite size values obtained from the *FWHM*_corr_ values of the observed reflections using the Scherrer equation with the Scherrer coefficient *K*_Scherrer_ = 0.94 [34]. All the calculations were performed after correcting the XRD patterns for the contribution of Cu-*K_α_*_2_ radiation using the EVA [30] program.

Magnetometric and magneto-optical methods were applied to study the static magnetic properties of BaFe_12_O_19_/Al_2_O_3_ nanostructures. A vibrating magnetometer (Lake Shore Cryotronics, Westerville, OH, USA) and a PPMS-9 complex (Quantum Design Inc., San Diego, CA, USA) were used to measure the magnetization curves using the magnetometric technique of VSM with a magnetic field **H** oriented, both along the normal to the film plane and for various orientations in the plane of the structure. During the VSM measurements, the magnetic field magnitude *H* was varied in the range from +20 to −20 kOe.

Utilizing a polarimetric setup at a wavelength of *λ* = 405 nm, the field dependences of the PMOKE at normal incidence were measured. The orientation of the magnetic field **H** was chosen either normal to the plane (**H**_out-of-plane_) or in the plane of the structure (**H**_in-plane_) with various azimuths *θ*. The magnetic field magnitude was varied in the range from +25 to −25 kOe. By the slow scanning of the magnetic field, the rotation of the plane of polarization of the reflected light was registered. A Faraday cell at a frequency of *f*~400 Hz with an amplitude of ~1° was used to modulate the polarization of the incident (or reflected) light in order to increase the sensitivity of measuring the rotation of the polarization plane.

Thus, the methods used in the work made it possible to conduct experiments on the growth of films by using the LMBE method on substrates of different sapphire cuts using different growth protocols, and also to study the crystal structure of the films, the mutual orientation of the crystal structure of the substrate and the film, as well as the magnetic properties of the films, including their magnetic anisotropy, and hysteresis loops in different geometries.

## 3. Results and Discussion

### 3.1. Surface Morphology

**Figure 1:**
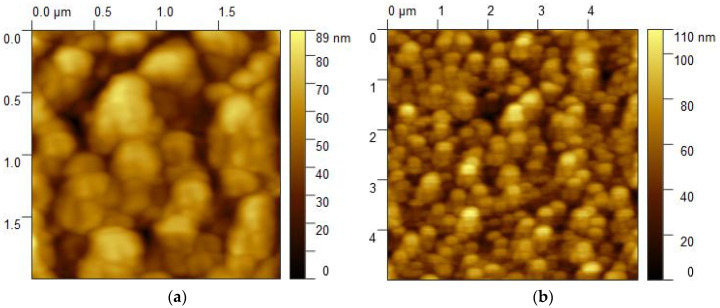
(**a**,**b**) AFM images of annealed sample #1 (BaFe_12_O_19_/Al_2_O_3_ (1–102) at different scales.

An AFM image of the surface of an annealed sample #1 (BaFe_12_O_19_/Al_2_O_3_ (1–102)) with a thickness of 50 nm showed that the film consists of nanocrystallites with lateral sizes of ~(200–300) nm (Figure 1a,b). The crystallites are densely packed, but between some, there are quite deep voids, reaching almost to the substrate. The average RMS roughness of such a surface is *RMS* = 15.8 nm over an area of 5 × 5 μm^2^. As compared to annealed BaM hexaferrite films grown on Al_2_O_3_ (0001) substrates, which consist of a set of nanocrystallites with clear faceting characteristic of crystals whose sixth-order axis is directed normal to the substrate plane [24], in the structure grown on an Al_2_O_3_ (1–102) substrate faceting of nanocrystallites, is not so clear. Nevertheless, some signs of faceting can be seen in Figure 1a. This may be due to the specific formation of hexaferrite nanocrystals on substrates in which the direction of the hexagonal axis is oriented at an angle to the plane of the structure.

### 3.2. RHEED Analysis

**Figure 2:**
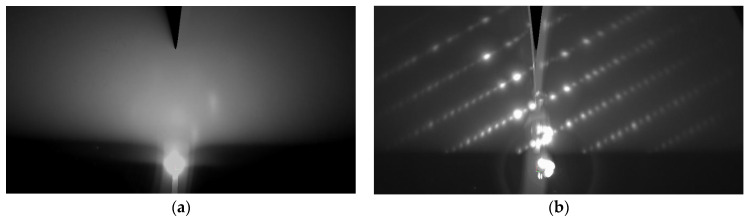
Cross-section of 3D RHEED of a sample #1 grown on Al_2_O_3_ (1–102) (**a**) before and (**b**) after annealing at 1000 °C. The 3D RHEED projections onto a plane are presented, in which the horizontal axes of (**a**,**b**) are parallel to the [−2–241]_BaM_ direction, and the vertical axes are parallel to [11–24]_BaM_.

Films grown on an Al_2_O_3_ (1–102) substrate (R-cut), as well as on an Al_2_O_3_ (0001) substrate (C-cut) immediately after the end of the growth process, exhibited very “hazy” RHEED diffraction patterns, which were difficult to interpret because of low signal/background ratio. However, after annealing in an air atmosphere, the crystalline quality of the film increased sharply, and a pattern consisting of rows of dots appeared on the RHEED (Figure 2). In the samples grown on Al_2_O_3_ (0001) substrates using a one-step protocol and annealed in air (samples #2 and #4), the electron scattering intensity distributions in reciprocal space were similar to those obtained in [24].

### 3.3. XRD Analysis

#### 3.3.1. Powder XRD

**Figure 3:**
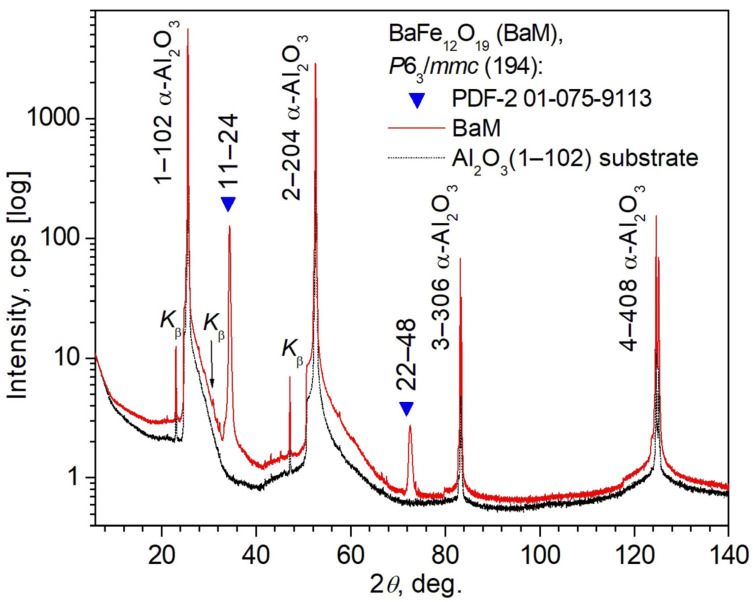
XRD patterns of the BaM film prepared on an Al_2_O_3_ (1–102) substrate (sample #1) and a virgin substrate on a logarithmic scale. The Miller-Bravais indices *hkil* are indicated for the observed reflections of the hexagonal BaM phase of the film and for reflections of the rhombohedral (in a hexagonal setting) α-Al_2_O_3_ phase of the substrate. The symbol *K_ß_* marks reflections arising from residual Cu-*K_ß_* radiation. The observed BaM reflections are shown by triangle symbols at the Bragg angles according to the PDF-2 card 01-075-9113. The space group of the BaM phase is indicated in the figure.

The measured XRD patterns of sample #1 prepared on an Al_2_O_3_ (1–102) substrate and a virgin Al_2_O_3_ (1–102) substrate are shown in Figure 3.

XRD patterns contain reflections of the α-Al_2_O_3_ phase (space group $R\bar{3}c$ (167) in a hexagonal setting, *a* = 4.7606(5) Å, *c* = 12.994(1) Å [25] at room temperature according to card 01-077-2135 of the PDF-2 powder database) from the substrate, corresponding to different orders of the 1–102 reflection in accordance with the orientation (1–102) of the α-Al_2_O_3_ substrate. Note that for the convenience of subsequent discussion, Figure 3 shows the Miller-Bravais indices *h*–*h*0*l* for the α-Al_2_O_3_ reflections of the substrate instead of the equivalent designation 0*h*–*h*0l adopted in the PDF-2 powder database (more precisely, Miller indices 0*hl* in the database).

In addition to the intense reflections from the α-Al_2_O_3_ substrate, the XRD pattern of sample #1 contains two reflections of the film that are also quite noticeable in regard to intensity (Figure 3), attributed to the BaM phase with the parameters of the hexagonal cell *a* = 5.875–5.90 Å and *c* = 23.007–23.218 Å according to various works for powders, single crystals, and films [14,35,36,37,38,39,40,41,42,43,44] (see Table S1 of Section S2 of the Supplementary Materials). Unfortunately, it is not possible to calculate the parameters of the unit cell of the BaM phase, since only two BaM reflections are observed, and not different ones, but corresponding to the first and second order of the same reflection *hkil* = 11–24. Nevertheless, the interplanar distances *d* = 2.6024(3) Å and 1.3012(1) Å, corresponding to the reflections from the film observed on the XRD pattern (Figure 3), agree quite well with the values *d* = 2.6154 Å–2.6301 Å and 1.3077 Å–1.3151 Å, corresponding, respectively, to the reflections 11–24 and 22–44 of the BaM phase for the different samples from Table S1 of Section S2 of the Supplementary Materials. The values of *d* for the studied sample #1 were calculated according to Bragg’s law from the Bragg angle 2*θ*_B_ after including the necessary angular corrections for zero shift and displacement. In the absence of data in PDF-2, the values of *d* were calculated by using the PowderCell program, version 2.4 [45], using the unit cell parameters of the BaM hexaferrite from the corresponding References [13,38,39,41,42,43,44].

It should be noted that the observed reflections from the film can also be attributed to the phases BaFeO_3–*δ*_ (*δ* ≈ 0.35 and 0.19), which are characterized by the same hexagonal space group *P*6_3_/*mmc* (194) like the BaM phase, and close unit cell parameters *a* = 5.779 Å–5.784 Å and *c* = 24.609 Å–24.63 Å [46,47] (see Supplementary Materials Table S1). In this case, the observed reflections can be indexed as 11–24 and 22–48 or 10–18 and 2 0 −2 16 reflections of the BaFeO_3–*δ*_ phase. The values *d* = 2.6207 Å–2.6229 Å and 1.3078 Å–1.3115 Å calculated from the unit cell parameters for these reflections of the phase are also in satisfactory agreement with the interplane distances indicated above for the corresponding observed reflections. However, the presence of a BaFeO_3–*δ*_ film with crystal lattice parameters very close to BaM seems unlikely, since, as a rule, the stoichiometry of substance transfer from the target to the substrate does not depend on the orientation of the substrate (except in the cases of a chemical reaction with the substrate), and for Al_2_O_3_ (0001) substrates, the chemical analysis of the film was carried out [24], as a result of which it was found that the stoichiometric composition of the film is close to BaM.

Thus, the BaM film of sample #1 exhibited the ideal effect of preferential orientation along the crystallographic direction [11–24]_BaM_, as a result of which only different orders of reflection 11–24 were observed, and reflections with other Miller-Bravais indices *hkil* were not observed, which indicates the absence of a polycrystalline component in this BaM film. This peculiarity distinguishes the BaM film of sample #1 grown on an Al_2_O_3_ (1–102) substrate from the BaM films in [24]. In [24] it was shown that for BaM films grown on an Al_2_O_3_ (0001) substrate, in addition to a large number of reflections of significant intensity 000l, a large number of weak reflections hkil were observed. This means that in these films, in addition to a large number of crystallites with the [0001]_BaM_ direction, located parallel to the [0001] normal of the Al_2_O_3_ (0001) substrate, there were a noticeable number of randomly located crystallites, as in a polycrystal.

Calculations using the graphical methods of linear profile analysis (Williamson-Hall plot (WHP) and size-strain plot (SSP) methods for the reflections of the observed pV-type), previously carried out for BaM films on Al_2_O_3_ (0001) substrates [24], showed the presence of crystallites with sizes *D* ≈ 50–145 nm at a nominal film thickness of *h* = 500 nm and noticeable microstrains *ε*_s_ ≈ 0.07–17%. In contrast, thin films with a thickness of *h* = 50 nm were characterized by the absence of microstrains (*ε*_s_ = 0) and crystallite sizes *D* ≈ 40 nm, comparable with the film thickness.

Unlike [24], where XRD patterns contained quite a lot (more than 10) of reflections with different Miller-Bravais indices *hkil* attributed to BaM, the number of BaM reflections for films on Al_2_O_3_ (1–102) was only two, and they were of the same type (two orders of reflection 11–24; see Figure 3). The estimates based on two observed BaM reflections in the zero microstrain model (*ε*_s_ = 0) according to the Scherrer equation gave an average crystallite size of *D*_0_ = 25(11) nm, i.e., a value comparable to that found in [24] for BaM films of the same nominal thickness (*h* = 50 nm) on Al_2_O_3_ (0001) substrates. The limited number of reflections did not allow the use of the WHP and SSP techniques with sufficient reliability to determine the possible presence of microstrains in the crystallites of the BaM film. To increase the reliability of the estimates, it is necessary to increase the number of the observed orders of the 11–24 reflection, for example, by using X-ray radiation with a shorter wavelength. Transmission electron microscopy could be applied to verify the size of crystallites, additionally. The use of these complementary techniques is planned in further studies.

So, the results of powder X-ray structural analysis in the present work and in [24] allow us to conclude that in films deposited on Al_2_O_3_ (1–102) and Al_2_O_3_ (0001) substrates via the LMBE method, using the growth and annealing parameters summarized in Table 1, a crystalline phase with the BaM hexaferrite structure grows. In the film of the sample #1, the [11–24] direction of the BaM phase is parallel to the normal [1–102] of the Al_2_O_3_ (1–102) substrate, and there are not enough BaM crystallites with other orientations to be detected by using powder XRD.

#### 3.3.2. Three-Dimensional XRD Reciprocal Space Mapping

**Figure 4:**
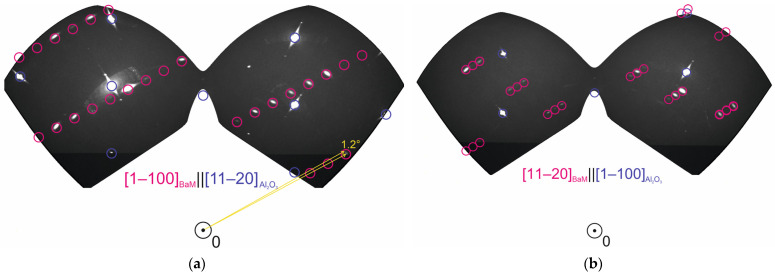

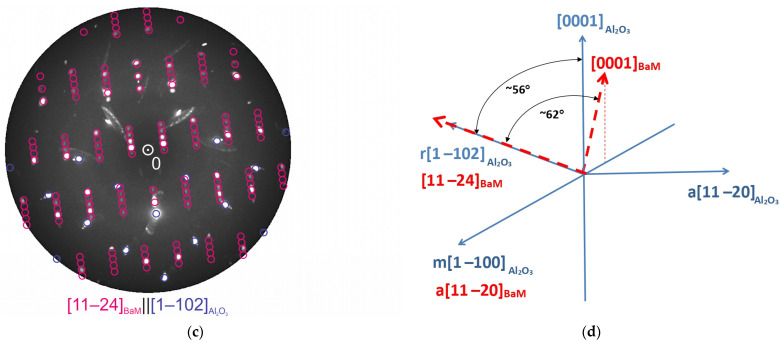
(**a**) Three cross-sections of reciprocal space taken by XRD from sample #1 for different crystallographic substrate orientations: (**a**) a[11–20], (**b**) m[1–100], and (**c**) r[1–102], where a, m, and r are the normals to A-, M-, and R-cuts of the Al_2_O_3_ substrate. Red circles are model positions for reflections from the BaM hexaferrite lattice. Blue circles correspond to the model positions for reflections from the Al_2_O_3_ substrate. White spots are observed XRD reflections from the substrate and BaM hexaferrite film. Yellow arrows in (**a**) indicate rotation of 1.2° around m [1–100] axis of BaM. The origin of coordinates on (**a**–**c**) is indicated by a circle with a dot in the center and marked with the number 0. (**d**) Schematic representation of substrate and film orientations.

For a detailed analysis of the crystal structure of the annealed BaM film grown on an Al_2_O_3_ (1–102) substrate, the method of 3D XRD reciprocal space mapping was used. Figure 4a shows three sections of the reciprocal space. The modeling of diffraction patterns shows that the cross-sections of the reciprocal space of BaM film/Al_2_O_3_ (1–102) substrate are well modeled by the BaM hexaferrite (space group *P*6_3_/*mmc* (194)) and α-Al_2_O_3_ (space group $R\bar{3}c$ (167)) lattices (see [14,25] and PDF-2 cards 01-075-9113 and 01-077-2135, respectively, as an example). All the reflections relate either to the substrate or to the BaM film, which proves that there are no other phases and textures in the structure (confirmed by the turns around the normal).

The epitaxial relations seemed nontrivial. As shown above using RHEED and powder XRD, the normal [1–102] of the Al_2_O_3_ (1–102) substrate was parallel to the [11–24] direction of BaM. Three-dimensional mapping confirmed this conclusion. In the film plane, the [11–20] Al_2_O_3_ direction was almost parallel to the [22–4–1]_BaM_ direction; however, there was a rotation of the film crystal lattice around the [1–100]_BaM_ axis by an angle of about 1.2°.

Thus, it can be argued that after annealing, BaM hexaferrite films of high crystalline quality are formed on the Al_2_O_3_ (1–102) substrate. Polycrystallinity was not observed, i.e., the film was single-crystalline. The hexagonal axis of the film was deviated from the normal to the film (and to the substrate) surface by an angle of *φ*~62° (Figure 4d).

It should be noted that during epitaxial growth, compensation of the lattice parameter mismatches between the substrate and film occurs in the interface layer in such a way that the elastic energy of the system is minimal. During the growth of the BaM hexaferrite film on Al_2_O_3_ (0001) substrates, such compensation occurs with the collinear orientation of the substrate and film axes, but even in this case, small variations of hexagonal axis orientation were observed [29]. During the growth on the R-cut substrates, the situation was somewhat more complicated, since the atomic planes oriented at an angle to the surface are conjugated at the interface. Therefore, some misalignment of the C_3_ axis of the substrate and the C_6_ axis of the film can be expected. As the results of the XRD data analysis show, in the case of R-cut, this misalignment is relatively small, and only a few degrees.

Thus, the comparison of AFM images, RHEED patterns of the as-grown and annealed films, as well as the analysis of the powder XRD patterns and 3D XRD reciprocal space mapping showed that after annealing in air on the R-cut substrate, an epitaxial film of BaM hexaferrite was obtained, in which the direction of the hexagonal axis was deviated by ~62° relative to the normal to the film surface (Figure 4d). This is in agreement with the results of the following magnetic measurements.

### 3.4. Magnetic Properties

#### 3.4.1. BaFe_12_O_19_/Al_2_O_3_ (1–102) (R-Cut) Heterostructures

Sample #1 was grown on an Al_2_O_3_ (1–102) substrate, in which the trigonal axis C_3_ of the Al_2_O_3_ crystal structure was inclined at an angle of ~56° from the normal to the (1–102) Al_2_O_3_ plane. As 3D XRD reciprocal space mapping has shown, the hexagonal axis in the film is directed at an angle of *φ*~62° to the sample normal, so it was expected that the EA of magnetization would also be directed at this angle to the substrate normal.

**Figure 5:**
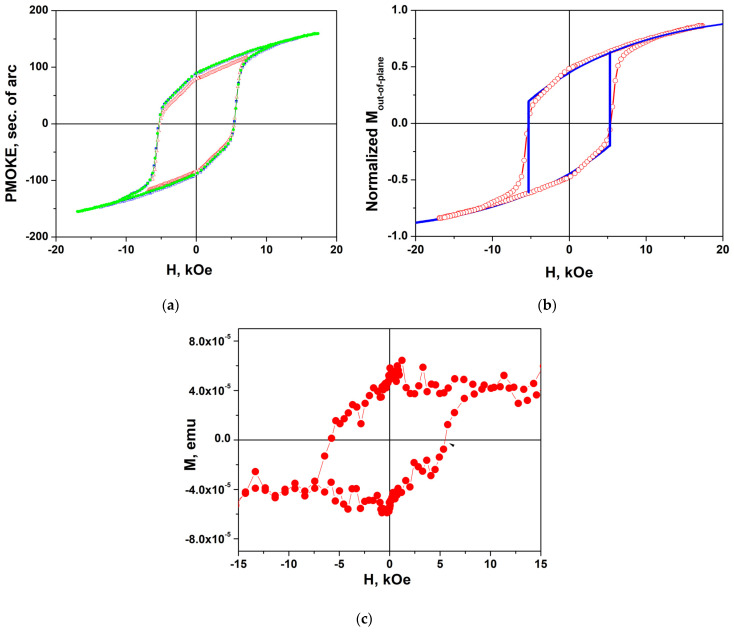
(**a**) PMOKE hysteresis loop in sample #1 for different maximal values of out-of-plane magnetic field (*H*^m^_out-of-plane_ = 7 kOe (red hollow triangles), 12 kOe (blue half-colored squares), and 17 kOe (green solid circles)). (**b**) Comparison of the experimental loop (red hollow circles) and that (blue solid line) calculated using the Stoner–Wohlfarth model for the values 4*πM*_s_ = 4.5 kG, *H*_a_ = 18 kOe, *φ* = 62°. (**c**) Magnetization curve *M*(*H*) measured using VSM after subtracting the linear part in *H* that appears in high fields caused by substrate magnetic susceptibility and film magnetization rotation. Lines connecting the symbols in (**a**–**c**) are guides for the eye.

Figure 5 shows the hysteresis loops measured by using the PMOKE (Figure 5a,b) and VSM (Figure 5c) methods in annealed sample #1 for an out-of-plane magnetic field.

It should be noted that the use of the PMOKE and VSM methods for studying BaM hexaferrite films has certain advantages over the known methods of magnetic force microscopy (MFM) [48] or superconducting quantum interference device (SQUID) magnetometry [49]. Since hexaferrite has a large magnetic moment, its measurement, even in thin films, does not require long-term signal accumulation, as in SQUID, and is easily detected using VSM. Measuring hysteresis loops using PMOKE at a wavelength of 405 nm has its own advantage associated due to the fact that, unlike magnetometric methods, the magnetic moment of the substrate does not appear in PMOKE loops, since even at a layer thickness of ~20 nm, the contribution of the substrate to the measured rotation of the plane of polarization is negligibly small. It should be noted that both VSM and PMOKE reflect the behavior of the bulk magnetization, which is important for microwave applications, while the MFM measurements provide information about the surface magnetization of films, which may differ from the bulk magnetization.

PMOKE loops proportional to *M*_out-of-plane_(*H*_out-of-plane_) differ significantly from the almost rectangular loops of the structures grown on Al_2_O_3_ (0001) substrates presented in [24]. In magnetic field regions before PMOKE jumps at *H*_c_ = ±5.2 kOe (i.e., from +20 kOe to −5.2 kOe or from −20 kOe to +5.2 kOe), strong reversible changes of PMOKE caused by the magnetization rotation are observed. Note that in the PMOKE measurements carried out at *λ* = 405 nm, the contribution of the substrate to the measured signal is negligible, and practically all changes of PMOKE are caused by the out-of-plane magnetization component *M*_out-of-plane_ of the film. In contrast, in the VSM measurements (Figure 5c), the contribution of the substrate to the measured signal is quite large. The loop shown in Figure 5c, obtained after subtracting the linear part in *H*, which is observed in high fields, is of the rectangular type.

A PMOKE hysteresis loop is well described by the Stoner–Wohlfarth model when taking into account the parameters characteristic of BaM hexaferrite—the magnetization value 4*πM*_s_ = 4.6 kG, the uniaxial anisotropy field *H*_a_ = 18 kOe, and the angle *φ* = 62° between the easy axis and the normal to the surface (Figure 5b). The jumps in the *M*_z_ component at *H* = ±*H*_c_ obviously associated with the appearance and movement of domain walls are shown in Figure 5c by the connecting lines running almost vertically. The details of the calculations using the Stoner–Wohlfarth model are described in Section S3 of Supplementary Materials (Figures S4–S7).

A jump-like change in the magnetization at *H* = ±*H*_c_ indicates that the film contains defects in the crystalline or magnetic structure, which counteract the nucleation of domains with the opposite magnetization sign and the motion of the domain walls. In the BaFe_12_O_19_/Al_2_O_3_ (0001) heterostructures with a hexaferrite structure close to ideal, in the absence of a magnetic field, the normal component of the magnetization is absent, since the film is easily broken down into the domains with the opposite magnetization sign [15]. Contrary to this, in the BaFe_12_O_19_/Al_2_O_3_ (0001) heterostructures obtained in [24], in which rectangular hysteresis loops are observed, the presence of defects is confirmed by the FMR spectra.

**Figure 6:**
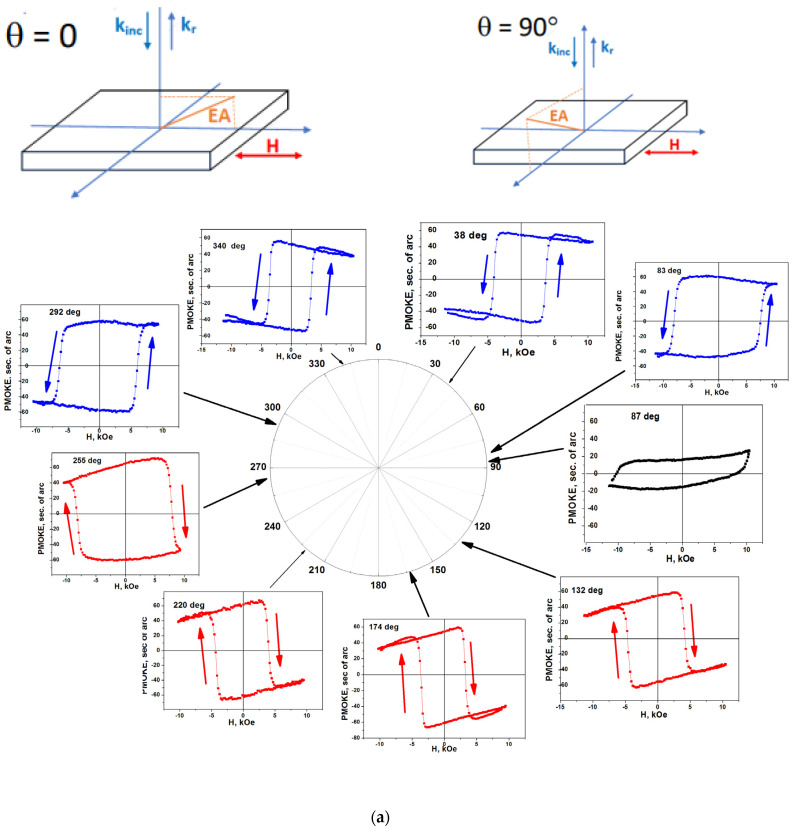

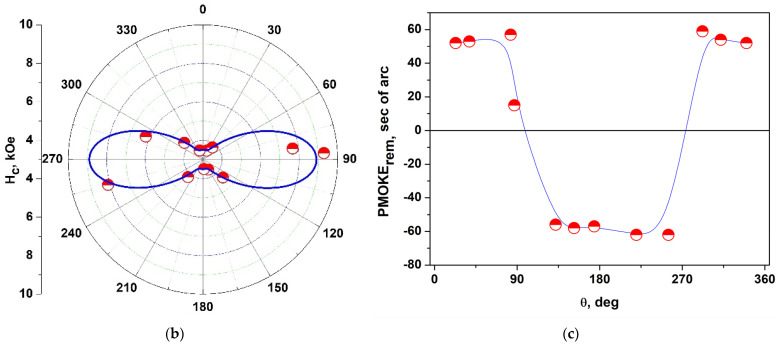
Sample #1. (**a**) Hysteresis PMOKE loops for various in-plane magnetic field **H**_in-plane_ orientations (red, blue, and black solid squares). Azimuths *θ* of in-plane magnetic field for the corresponding hysteresis loops are shown by arrows. Left and right insets in the top of (**a**) show mutual orientation of EA and magnetic field for *θ* = 0° and 90°. Blue (for *θ* = 38°, 83°, 292°, and 340°) and red (for *θ* = 132°, 174°, 220°, and 255°) symbols correspond to different sign of scalar product **uH,** where **u** is unit vector along EA. Black symbols (for *θ* = 87°) correspond to the orthogonal mutual orientation of **u** and **H**. Angular dependence of (**b**) the coercive field *H*_c_ and (**c**) the remnant value of PMOKE. Symbols (red half-colored circles) represent the experimental points. Lines connecting the symbols in the PMOKE loops in (**a**) and blue solid lines in (**b**) (thick line) and (**c**) (fine line) are provided as a guide for the eye.

Magnetic hysteresis loops were studied also for the in-plane orientation of the magnetic field. When using the PMOKE technique at normal incidence, the PMOKE magnetization curves give a change in the out-of-plane magnetization component *M*_out-of-plane_ in the in-plane magnetic field **H**_in-plane_. The orientation of **H**_in-plane_ in the plane of the structure is characterized by azimuth angle *θ*. Since the projection of the EA of magnetization onto the **H** direction depends on *θ*, then the hysteresis loops must also depend on *θ*. This is confirmed by the results of the experiment (Figure 6).

When the projection of the EA onto the film plane was orthogonal to the direction of the magnetic field (top right inset in Figure 6a), a very weak dependence of PMOKE on the magnetic field was observed, characterized by a large value of *H*_c_ and a small remnant value of PMOKE (Figure 6, panel *θ* = 87°). In the vicinity of such position, the hysteresis loops were very broad (Figure 6, panels *θ* = 83°, 255°, and 292°). When the projection of the EA was parallel to the orientation of the magnetic field (*θ* = 0°, left inset in Figure 6a), a loop with minimal coersive field *H*_c_ = 3.5 kOe and a large remnant value of PMOKE was observed (panels *θ* = 340° and 174°). The angle variation in *H*_c_ and remnant PMOKE are shown in Figure 6b,c. A strong increase in *H*_c_ and almost abrupt change in the sign of the remnant PMOKE values were observed at *θ* = 90° and 270°. Such behavior is explained by taking into account the changes in the mutual orientation of the EA and the magnetic field **H**_in-plane_. Just in the vicinity of these angles, the scalar product **M·H** strongly decreased, and the projection of a unit vector **u**, parallel to the EA, onto the positive magnetic field **H**_in-plane_ changed sign.

**Figure 7:**
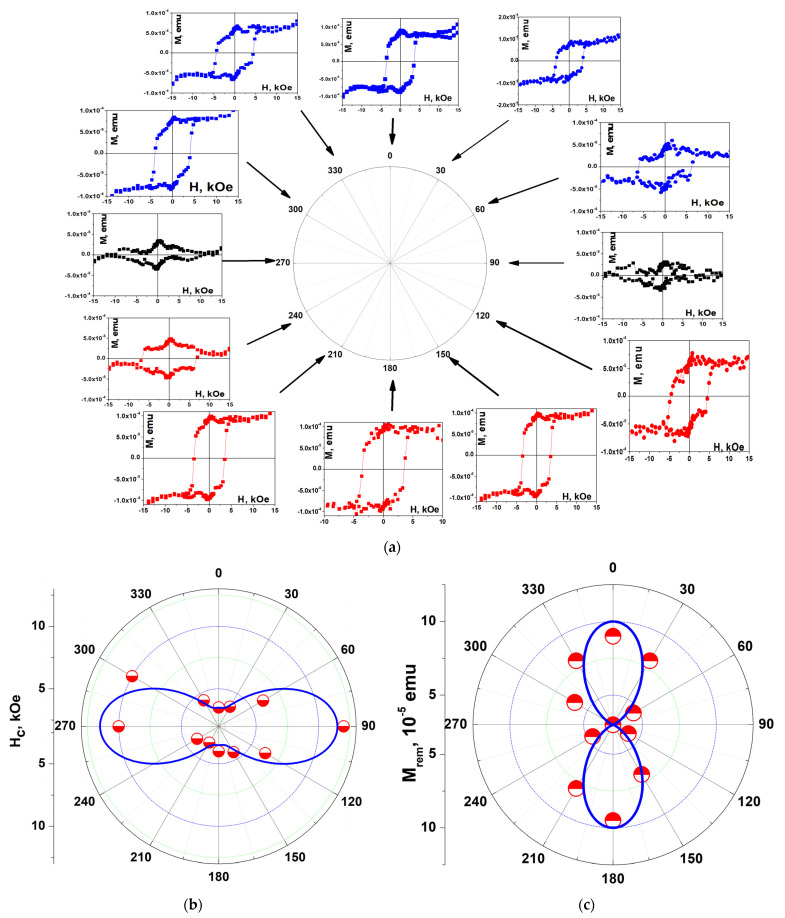
(**a**) Hysteresis loops in sample #1 measured by using VSM for various azimuths *θ* of in-plane magnetic field **H**_in-plane_ (red, blue, and black solid squares). The loops were obtained after subtracting the linear part in *H*, which appears in high fields and is caused mainly by the substrate magnetization. Blue (for *θ* = 0°, 30°, 60°, 300°, and 360°) and red (for *θ* = 120°, 150°, 180°, 210°, and 240°) symbols in (**a**) correspond to different sign of scalar product **uH**. Black symbols (for *θ* = 90° and 270°) correspond to orthogonal mutual orientation of **u** and **H**. Angular dependence of (**b**) the coercive field *H*_c_ and (**c**) the remnant magnetization *M*_rem_. The experimental points in (**b**,**c**) are shown by red half-colored circles. Lines connecting the symbols in the PMOKE loops in (**a**) and thick blue solid lines in (**b**,**c**) are provided as a guide for the eye.

The magnetization curves for an in-plane magnetic field measured by using the VSM method are associated with the changes in magnetization *M*_in-plane_(*H*_in-plane_) and also strongly depend on the azimuth *θ* (Figure 7). As noted above, the magnetization measured by using the VSM method is due to the magnetization of both the BaM hexaferrite film and the Al_2_O_3_ substrate. Subtracting the linear part in the magnetic field observed in the high fields from the experimentally measured magnetization curves eliminates the contribution of the substrate as well as the contribution of the rotation of the film magnetization. Nevertheless, strong changes in the hysteresis loop are evident in Figure 7. For *θ* = 90° and 270°, when the EA is perpendicular to the magnetic field, the magnetization curves are associated only with the magnetization rotation caused by **H**_in-plane_, and pronounced loops are practically absent. Strong hysteresis loops were observed at *θ* = 0° and 180°, when the absolute value of the projection of the EA on **H** is maximal.

Thus, in the epitaxial films grown on the R-cut (1–102) sapphire substrates, the easy axis of magnetization was deviated from the normal to the surface, which led to the peculiarities of the magnetization curves compared to the films grown on the C-cut (0001) substrates. The hysteresis loop of the *M*_out-of-plane_ component versus the *H*_out-of-plane_ field contains parts associated with the magnetization rotations, and in the case of the in-plane orientation of the magnetic field, the field dependences of the *M*_out-of-plane_ and *M*_in-plane_ components and loop parameters, such as the coercive field *H*_c_ and remnant magnetization *M*_rem_, depend on the azimuth *θ* of the field in the plane, demonstrating 180° periodicity, which is due to the rotation of the EA around the normal to the surface. In the absence of a magnetic field, a single-domain state with an inclined orientation of magnetization is realized in the films.

#### 3.4.2. Two-Stage Growth Protocol (Al_2_O_3_ (0001) (C-Cut) Substrates

As noted above, the crystal structure of BaM hexaferrite and the corresponding magnetic properties in the grown structures appeared after their annealing in air (oxygen pressure *p* = 0.06 mBar) at *T*_ann_ = 1000 °C. The annealing of sample #6, grown at *T*_gr_ = 700 °C and *p* = 0.05 mBar, at an oxygen pressure *p* = 0.5 mBar directly in the growth chamber at a temperature *T*_ann_ = 1000 °C for an hour did not lead to the appearance of magnetization. We also note that the annealing of the samples in a nitrogen atmosphere at *T*_ann_ = 950 °C led to the complete degradation of the film, accompanied by a change in its color (Supplementary Materials Figure S2c). These observations indicate that a sufficiently high oxygen pressure and temperature *T*_ann_~1000 °C are necessary for the formation of the BaM hexaferrite layer. Under these conditions, BaM hexaferrite crystallizes even in the presence of nitrogen contained in the air.

An increase in the film thickness to 300–500 nm was accompanied by a change in the shape of the hysteresis loop, in particular, an increase in the range of fields in which the sign of magnetization changed [24]. This indicates the accumulation of crystalline and magnetic inhomogeneities in them. When thin layers (*h* ≤ 50 nm) are grown, the lattice parameters of the film approximately correspond to the substrate lattice parameters. This correspondence is also preserved with a decreasing temperature. In contrast, in thicker films (*h* ≥ 100 nm), the lattice parameters of the film relax and tend to their values in the bulk single crystal. When the temperature is decreased, the difference in the thermal expansion coefficients results in thermal stress, which in some cases, can cause film cracking. Even if cracking is absent, the presence of inhomogeneities leads to a spread of magnetic parameters, such as the energy of domain boundary nucleation, the velocity of spin wave propagation, etc. The variation in the magnetic parameters is accompanied by a change in the loop shape, in particular, the appearance of inclined rather than vertical wings of the hysteresis loop, accompanying a change in the magnetization sign. In addition, the presence of such inhomogeneity can manifest itself in undesirable attenuation of the spin waves propagating in the film. For this reason, films with a sharp jump in magnetization switching are preferable.

To overcome these shortcomings, experiments were undertaken using a two-stage protocol. In this case, the structure was extracted after the first growth cycle from the growth chamber, annealed in a muffle furnace, and then grown on a second film on the already formed hexaferrite/sapphire heterostructure.

**Figure 8:**
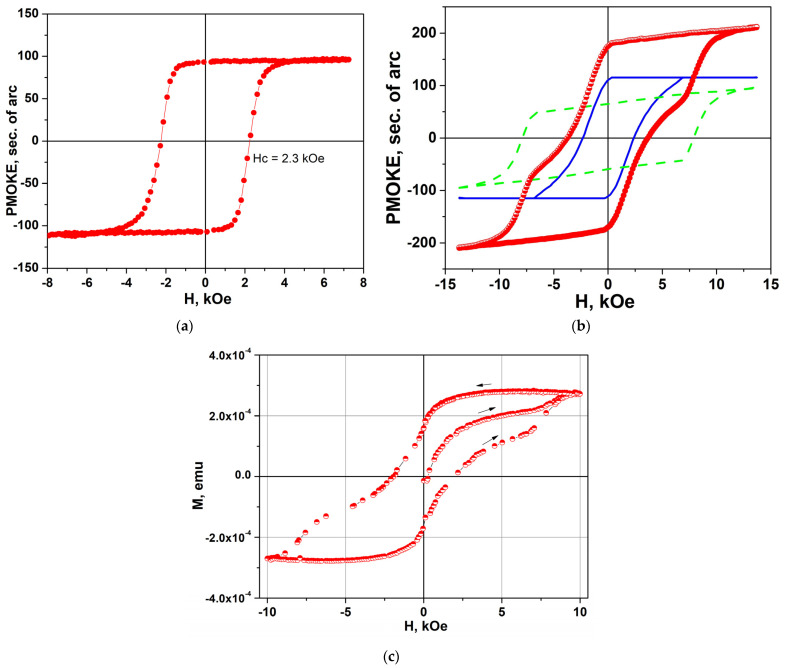
PMOKE hysteresis loop (**a**) in sample #2 after annealing (first stage of protocol) and (**b**) in sample #3 (second stage). Blue solid and green dashed lines in panel (**b**) show the decomposition of the loop into two loops. (**c**) Magnetization curve of sample #3 measured by using VSM. Red solid circles in (**a**) and red half-colored circles in (**b**,**c**) are experimental points. Lines connecting the symbols in (**a**–**c**) are a guide for the eye.

Sample #3 was obtained by growing a 150 nm thick film on an already grown and annealed 20 nm thick film #2 and annealed in air in accordance with the two-stage protocol. The PMOKE hysteresis loop in annealed sample #2 (the first stage of the protocol) was almost rectangular with the ratio *M*_rem_/*M*_s_ ≈ 1 and the coercive field *H*_c_ ≈ 2.3 kOe (Figure 8a). Growing a second layer of 150 nm thickness on it was not accompanied by the appearance of magnetization before the following annealing. This means that growing a layer on a substrate with an already formed BaM structure is insufficient for the formation of a crystalline structure in the second layer. Hysteresis loops appear only after the second annealing in air (Figure 8b,c). Apparently, the key parameter for the appearance of the BaM hexaferrite structure is a sufficient oxygen pressure to exclude the appearance of oxygen vacancies.

Note that the hysteresis loop of sample #3 measured by using PMOKE (Figure 8b) or VSM (Figure 8c) has a complex shape and can be represented as a sum of two hysteresis loops with different *H*_c_ values. For the PMOKE case, the result of the hysteresis loop decomposition is shown in Figure 8b by blue solid and green dashed lines. The coercive fields of narrow and wide hysteresis are *H*_c_ ≈ 2.3 kOe and 3.7 kOe, respectively. The value of *H*_c_ = 2.3 kOe practically coincides with the value of *H*_c_ of sample #2, which was used as a heteroepitaxial substrate for the preparation of sample #3. The value of *H*_c_ ≈ 3.5 kOe (or higher) is typical of films with a thickness of 200–300 nm [24]. It is important to note that the PMOKE signal measured at the wavelength *λ* = 405 nm is due to the magnetization of the layer less than 50 nm thick, i.e., it is related to the magnetization of the second layer only. This indicates that at least two magnetizations are present in this layer, one strongly coupled to the magnetization of the first hexaferrite layer and the other unrelated to it. This was also confirmed by the VSM measurements, since 90% of the measured magnetic moment in sample #3 was caused by the magnetization of the second layer due to the differences in the thicknesses of the layers (20 nm and 150 nm, respectively). Thus, the growth of a thicker film on a thin one can lead to the strong inhomogeneity of the magnetization of the structure.

**Figure 9:**
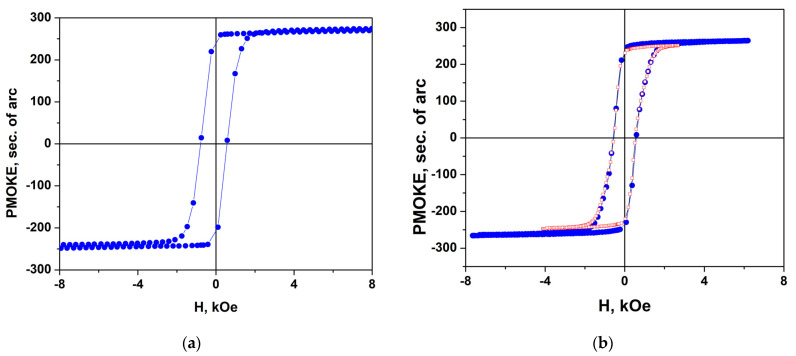
PMOKE hysteresis loop (**a**) in sample #4 (first stage of protocol) and (**b**) in sample #5 (second stage). Red (hollow circles) and blue (solid circles) symbols in (**b**) correspond to experimental points obtained using various maximal values of magnetic field. Lines connecting the symbols in (**a**,**b**) are a guide for the eye.

Sample #5 was also grown using a two-stage protocol. The 50 nm thick upper film was grown on annealed 50 nm thick structure #4 and annealed in air. The hysteresis loop of sample #4 was almost rectangular with a coercivity *H*_c_~0.7 kOe (Figure 9a). In annealed structure #5, the loop was characterized by coercive field *H*_c_~0.6 kOe and was quite rectangular (Figure 9b).

A summary of the parameters of the hysteresis loops in out-of-plane geometry (*M*_out-of-plane_(*H*_out-of-plane_)) for BaM-samples #1–5 and samples BaM/Al_2_O_3_ (0001) with thicknesses of 50 nm, 250 nm, and 500 nm from Ref. [24] are presented in Table 2.

It is evident from Table 2 that for out-of-plane geometry (*M*_out-of-plane_(*H*_out-of-plane_)) in film with an inclined easy axis of magnetization (sample #1) the ratio *M*_rem_/*M*_sat_ is significantly smaller, and the saturation field *H*_sat_ is significantly larger than in film #2 with an out-of-plane easy axis orientation. Such differences can be clearly attributed to the different mechanisms of the magnetization process in films with different orientations of the easy magnetization axis. If in sample #2 the magnetization reversal goes only though domain wall creation and movement, in sample #1 the mechanism of magnetization rotation plays an important role.

Films thicker than 100 nm (#3, Loop 2, #8963 [24], and #9001B [24]) grown on Al_2_O_3_ (0001) substrates have a significantly larger *H*_sat_ value compared to analogous films with a thickness of ~50 nm (#2, #4, and #8948 [24]). Such difference reflects less rectangularity of the hysteresis loops in thick films as compared to thin. The reasons for such change in hysteresis loop shape may be attributed to the higher inhomogeneity of thick films due to the relaxation of the mismatch between the lattice parameters as mentioned above (see Section 3.4.2).

In film #5 (*h* = 100 nm), obtained using a two-stage growth protocol, all the parameters of the hysteresis loop coincide with the parameters of film #5 with a thickness of 50 nm, which was used as a substrate for growing the second layer. It can be assumed that this is due to the fact that the second layer grows on the already formed BaM crystal structure modified by the substrate, and since the thickness of the second layer is small (50 nm), the relaxation of the lattice parameter mismatch does not occur during the growth process.

Thus, the study of the two-stage growth protocol showed the following:

(1) The growth of the second layer on a well-formed structure of BaM hexaferrite without subsequent annealing does not lead to the appearance of a hexaferrite crystal structure of the upper layer.
(2) The growth of a thick layer on an annealed thin layer of BaM hexaferrite is accompanied after annealing by the appearance of a complex hysteresis loop, in which the peculiarities of the hysteresis loops of both the thin and thick layers are visible.
(3) Growing relatively thin films (thickness *h* = 50 nm) using a two-stage protocol is not accompanied by the deterioration of the magnetic hysteresis loops. This suggests that growth using a multi-stage protocol will allow obtaining thick, high-quality films with narrow rectangular loops.

As a result of the experiments, it was shown that BaFe_12_O_19_ films epitaxially grown by using the LMBE method on R-cut sapphire substrates after annealing have a hexaferrite crystal structure with a deviation of the hexagonal axis and easy magnetization axis from the normal to the surface by an angle of ~60°. This deviation results in the appearance of specific magnetic hysteresis loops in various experimental geometries and the implementation of an inclined orientation of the magnetization in the absence of a magnetic field. The use of a two-stage growth protocol showed the possibility of increasing the thickness of the magnetic layer without a significant decrease in the homogeneity of the film.

## 4. Conclusions

As shown in [15], to fabricate planar microwave devices operating at frequencies of up to 100 GHz, thin-film magnetic materials with magnetization oriented normally or at an angle to the plane of the film in the absence of a magnetic field and possessing a large magnetic moment sufficient for effective excitation and reception of spin waves are needed. Apparently, one of the best methods for achieving this goal is the LMBE method, which allows the obtaining of crystalline epitaxial films, for example, BaM hexaferrite films, on various substrates. In the presented work, it is shown that in order to increase the thickness of epitaxial BaM hexaferrite films obtained by using the LMBE method without losing their magnetic characteristics, such as the degree of rectangularity of magnetic hysteresis loops and the value of magnetization, a multi-stage growth protocol can be used, in which the growth of thin (~50 nm) layers alternates with their annealing in an air atmosphere at a temperature *T*_ann_ = 1000 °C. This was demonstrated for BaM hexaferrite films grown on Al_2_O_3_ (0001) substrates, which gives grounds to hope for obtaining structures with a large magnetic moment, consisting of many high-quality layers of BaM hexaferrite.

Another important result of the work is the production of an epitaxial film of BaM hexaferrite on a R-cut Al_2_O_3_ (1–102) sapphire substrate. After subsequent annealing in air, the BaM hexaferrite crystal structure with the direction of the sixth-order axis and the easy magnetization axis oriented at an angle of *φ*~62° to the film surface normal was realized in the film. After applying and removing the saturating magnetic field, a single-domain state with the magnetization orientation close to the EA direction was realized in the film. In such films, a significant reversible change in the normalized out-of-plane magnetization component *M*_out-of-plane_/*M*_s_ from 0.18 to 0.88 is possible in magnetic fields *H*_out-of-plane_ from +20 kOe to –5 kOe, which can be significant for the development of various microwave devices.

It would be of interest to investigate the influence of an out-of-plane electric field on the orientation of the EA and, consequently, on the orientation of the magnetization in such films. As shown by the previous studies on the electromagneto-optical effect in the epitaxial films of yttrium ferrite garnet, which are also characterized by an inclined orientation of the EA, the changes in the EA orientation can depend linearly on the electric field, even if both the substrate and the film have an inversion center. The appearance of a linear magnetoelectric effect in this case is associated with the violation of the inversion operation symmetry in the vicinity of the interface [50].

It seems that the prospects for the development of this work may also be associated with the study of ferromagnetic resonance spectra, both in the presence and in the absence of a magnetic field, as well as optimizing the LMBE growth conditions of BaM hexaferrite films to avoid an ex situ annealing procedure.

## Figures and Tables

**Table 1:** Sample number #, film thickness *h*, growth temperature *T*_gr_, oxygen pressure *p*, annealing time *τ*_ann_, annealing temperature *T*_ann_, and Al_2_O_3_ substrate orientation.

# Sample Number	Film Thickness *h*, nm	Growth Temperature *T*_gr_, °C	Oxygen Pressure *p*, mBar	Annealing Time *τ*_ann_, min; Annealing Temperature *T*_ann_*,* °C	Al_2_O_3_ Substrate	Number of Growth Experiment
1	50 ± 2	700	0.06	60; 1000	(1–102) ^a^	2
2	20 ± 1	700	0.06	60; 1000	(0001)	1
3	150 (total thickness 20 + 150 = 170 ± 2)	700	0.06	60; 1000	(0001)	1
4	50	700	0.06	30; 1000	(0001)	1
5	50 (total thickness 50 + 50 = 100 ± 3)	700	0.06	30; 1000	(0001)	1
6	50 ± 2	700	0.05 ^b^	60; 1000 ^b^	(0001)	2

^a^ equivalent to (01–12) or (10–12); ^b^ annealing in the growth chamber in an oxygen atmosphere at a pressure of *p* = 0.5 mBar.

**Table 2:** Parameters of hysteresis loops (*M*_out-of-plane_ as a function of *H*_out-of-plane_) in samples prepared by using one-stage and two-stage protocols. Sample number #, Al_2_O_3_ substrate orientation, BaM film thickness *h*, coercive field *H_c_*, saturation field *H*_sat_, *M_rem_*/*M_sat_* ratio (self-bias), type of growth protocol. Parameters of Loop 1 and Loop 2 were estimated from decomposition of hysteresis loop of sample #3 (Figure 8).

# Sample Number	Al_2_O_3_ Substrate Orientation	*h*, nm	*H*_c_, kOe	*H*_sat_, kOe	*M*_rem_/*M*_sat_, %	Growth Protocol
1 ^a^	(1–102)	50 ± 2	5.4	~20	~50	one-stage
2	(0001)	20 ± 1	2.3 ± 0.1	3.9 ± 0.1	97 ± 1	one-stage
3	(0001)	170 ± 2	3.7 ± 0.1	11.4 ± 0.2	83 ± 2	two-stage
Loop 1	(0001)	–	2.3 ± 0.1	6.8 ± 0.2	97 ± 1	–
Loop 2	(0001)	–	7.9 ± 0.1	11.3± 0.1	72 ± 2	–
#8948B ^b^ from Ref. [24]	(0001)	50 ± 2	2.1 ± 0.1	5.0 ± 0.2	98 ± 2	one-stage
#8963B from Ref. [24]	(0001)	250 ± 3	3.5 ± 0.1	12.4 ± 0.2	94 ± 2	one-stage
#9001B ^b^ from Ref. [24]	(0001)	500 ± 4	7.0 ± 0.2	17.4 ± 0.3	84 ± 2	one-stage
4	(0001)	50 ± 2	0.7 ± 0.1	1.8 ± 0.1	90 ± 2	one-stage
5	(0001)	100 ± 3	0.6 ± 0.1	1.8 ± 0.1	94 ± 2	two-stage

^a^ XRD pattern (Cu-*K_α_*, up to 2*θ* = 140°) contains only two orders of the BaM reflection 11–24, i.e., a strong preferential orientation along [11–24]_BaM_, coinciding with the [1–102] crystallographic direction of the Al_2_O_3_ (1–102) substrate, is manifested. ^b^ XRD pattern (Cu-*K_α_*, up to 2*θ* = 140°) contains different orders of 0001 BaM reflection and a lot of reflections with different Miller-Bravais indices *hkil*, i.e., in the BaM film there is a preferential orientation along [0001]_BaM_ (coinciding with the crystallographic direction [0001] of the Al_2_O_3_ (0001) substrate) and a lot of randomly oriented other BaM crystallites.

## Data Availability

Data are contained within the article or Supplementary Materials.

## Supplementary Materials

The following supporting information can be downloaded at https://www.mdpi.com/article/10.3390/nano14231883/s1, Section S1: Optical photos of samples; Figure S1: Optical image of sample #1 (thickness *h* = 50 nm), (**a**) first and (**b**) second growth experiment; Figure S2: Optical image of sample (**a**) #2 (thickness *h* = 20 nm, (**b**) #3 (*h* = 170 nm), and (**c**) structure #6 (*h* = 50 nm) annealed in nitrogen atmosphere at *T*_ann_ = 950 °C; Figure S3: Optical image of structure #4 (thickness *h* = 50 nm); Section S2: Powder XRD; Table S1: Unit cell parameters (*a*, *c*) and volume (*V*) and interplane distances *d* of reflections 11–24 and 22–48 of BaM and BaFeO_3–δ_ phases according to current work and literature data; Section S3: Calculations in Stoner–Wolfarth model; Figure S4: Schematic drawing of coordinate system, orientation of easy axis of magnetization (EA), magnetization *M*, and in-plane magnetic field *H* for (**a**) *θ* = 0°, (b) *θ* = 90°, and (**c**) out-of-plane orientation of magnetic field; Figure S5: Experimental (blue symbols) and calculated (red dashed and solid lines) *M*_out-of-plane_(*H*_in-plane_) dependence for *θ* = 0°; Figure S6: Magnetic field dependences of *m_x_*, *m_y_*, and m_z_ for orientation of in-plane magnetic field **H**_in-plane_ along *Y*-axis and orientation of EA in *XZ*-plane (*θ* = 90°); Figure S7: Experimental (blue symbols and blue thin solid line are a guide for eye) and calculated (red thick dashed and solid lines) dependence *M*_out-of-plane_(*H*_out-of-plane_); References [1–14] cited in the Supplementary Materials are present in the main text of the article: [14,37,38,39,40,41,42,43,44,45,46,48,49].

## References

[1] Enerdata, Grenoble, France. https://www.enerdata.net/publications/executive-briefing/between-10-and-20-electricity-consumption-ict-sector-2030.html.

[2] Kruglyak V.V., Demokritov S.O., Grundler D. (2010). Magnonics. J. Phys. D. Appl. Phys..

[3] Grundler D. (2016). Nanomagnonics. J. Phys. D. Appl. Phys..

[4] Nikitov S.A., Kalyabin D.V., Lisenkov I.V., Slavin A.N., Barabanenkov Y.N., Osokin S.A., Sadovnikov A.V., Beginin E.N., Morozova M.A., Sharaevsky Y.P. (2015). Magnonics: A new research area in spintronics and spin wave electronic. Phys. Uspeki..

[5] Serga A.A., Chumak A.V., Hillebrands B. (2010). YIG magnonics. J. Phys. D. Appl. Phys..

[6] Onbasli M.C., Kehlberger A., Kim D.H., Jakob G., Kläui M., Chumak A.V., Hillebrands B., Ross C.A. (2014). Pulsed laser deposition of epitaxial yttrium iron garnet films with low Gilbert damping and bulk-like magnetization. APL Mater..

[7] Sokolov N.S., Fedorov V.V., Korovin A.M., Suturin S.M., Baranov D.A., Gastev S.V., Krichevtsov B.B., Maksimova K.Y., Grunin A.I., Bursian V.E. (2016). Thin yttrium iron garnet films grown by pulsed laser deposition: Crystal structure, static, and dynamic magnetic properties. J. Appl. Phys..

[8] Yoshimoto T., Goto T., Shimada K., Iwamoto B., Nakamura Y., Uchida H., Ross C.A., Inoue M. (2018). Static and Dynamic Magnetic Properties of Single-Crystalline Yttrium Iron Garnet Films Epitaxially Grown on Three Garnet Substrates. Adv. Electron. Mater..

[9] Rana G., Dhiman P., Kumar A., Vo D.-V.N., Sharma G., Sharma S., Naushad M. (2021). Recent advances on nickel nano-ferrite: A review on processing techniques, properties and diverse applications. Chem. Eng. Res. Des..

[10] Hao A., Ning X. (2021). Recent Advances in Spinel Ferrite-Based Thin Films: Synthesis, Performances, Applications, and Beyond. Front. Mater..

[11] Dixit G., Singh J.P., Srivastava R.C., Agrawal H.M., Chaudhary R.J. (2012). Structural, magnetic and optical studies of nickel ferrite thin films. Adv. Mat. Lett..

[12] Liu Y., Zhou P., Regmi S., Bidthanapally R., Popov M., Zhang J., Zhang W., Michael R., Zhang T., Gupta A. (2022). Strain induced anisotropy in liquid phase epitaxy grown nickel ferrite on magnesium gallate substrates. Sci. Rep..

[13] Krichevtsov B.B., Suturin S.M., Korovin A.M., Kaveev A.K., Bursian V.E., Cuňado J.L.F., Sokolov N.S. (2022). Diffused magnetic transitions in NiFe_2_O_4_/SrTiO_3_(001) epitaxial heterostructures. J. Magn. Magn. Mater..

[14] Sharma P., Rocha R.A., Medeiros S.N., Hallouche B., Paesano A. (2007). Structural and magnetic studies on mechanosynthesized BaFe_12_–*_x_*Mn*_x_*O_19_. J. Magn. Magn. Mater..

[15] Harris V.G. (2012). Modern Microwave Ferrites. IEEE Trans. Magn..

[16] Harris V.G., Chen Z., Chen Y., Yoon S.D., Sakai T., Gieler A., Yang A.F., He Y., Ziemer K.S., Sun N.X. (2006). Ba-hexaferrite films for next generation microwave devices, invited. J. Appl. Phys..

[17] Pullar P.C. (2012). Hexagonal ferrites: A review of the synthesis, properties and applications of hexaferrite ceramics. Prog. Mater. Sci..

[18] Singh V.P., Jasrotia R., Kumar R., Raizada P., Thakur S., Batoo K.M., Singh M. (2018). A Current Review on the Synthesis and Magnetic Properties of M-Type Hexaferrites Material. World J. Condens. Matter Phys..

[19] Tsuyoshi K. (2012). Magnetoelectric Hexaferrites. Annu. Rev. Condens. Matter Phys..

[20] Zahid M., Siddique S., Anum R., Shakir M.F., Nawab Y., Rehan Z.A. (2021). M-Type Barium Hexaferrite-Based Nanocomposites for EMI Shielding Application: A Review. J. Supercond. Nov. Magn..

[21] Mohammed I., Mohammed J., Kende A.U., Mohammed W.A., Aliero Y.A., Magawata U.Z., Umar A.B., Srivastava A.K. (2023). Review on Y-type hexaferrite: Synthesis, characterization and properties. Appl. Surf. Sci. Adv..

[22] Mørch M.I., Ahlburg J.V., Saura-Múzquiz M., Eikeland A.Z., Cristensen M. (2019). Structure and magnetic properties of W-type Hexaferrites. IUCrJ.

[23] Coey J.M.D. (2020). Perspective and Prospects for Rare Earth Permanent Magnets. Engineering.

[24] Krichevtsov B., Korovin A., Suturin S., Levin A.A., Lobov I., Telegin A., Badalyan A., Sakharov V., Serenkov I., Dorogov M. (2023). Structural, Magnetic, and Magneto-Optical Properties of Thin Films of BaM Hexaferrite Grown by Laser Molecular Beam Epitaxy. Materials.

[25] Tsirelson V.G., Antipin M.Y., Gerr R.G., Ozerov R.P., Struchkov Y.T. (1985). Ruby structure peculiarities derived from X-ray data. Localization of chromium atoms and electron deformation density. Phys. Status Solidi A.

[26] Hylton T.L., Parker M.A., Coffey K.R., Howard J.K. (1993). Properties of epitaxial Ba hexaferrite thin films on A, R, and C plane oriented sapphire substrates. J. Appl. Phys..

[27] Yoon S.D., Vittoria C. (2003). Microwave and magnetic properties of barium hexaferrite films having the c-axis in the film plane by liquid phase epitaxy technique. J. Appl. Phys..

[28] Zhang X., Meng S., Song D., Zhang Y., Yue Z., Harris V.G. (2017). Epitaxially grown BaM hexaferrite films having uniaxial axis in the film plane for self-biased devices. Sci. Rep..

[29] Borisov P., Alaria J., Yang T., McMitchell S.R.C., Rosseinsky M.J. (2013). Growth of M-type hexaferrite thin films with conical magnetic structure. Appl. Phys. Lett..

[30] Bruker AXS (2019). Karlsruhe, Diffrac. Suite Eva Version 5.1.0.5.

[31] Fawcett T.G., Kabekkodu S.N., Blanton J.R., Blanton T.N. (2017). Chemical analysis by diffraction: The Powder Diffraction File™. Powder Diffr..

[32] Langford J.I., Cernik R.J., Louer D. (1991). The Breadth and Shape of Instrumental Line Profiles in High-Resolution Powder Diffraction. J. Appl. Crystallogr..

[33] Rehani B.R., Joshi P.B., Lad K.N., Pratap A. (2006). Crystallite size estimation of elemental and composite silver nano-powders using XRD principles. Indian. J. Pure Appl. Phys..

[34] Scherrer P. (1918). Bestimmung der Grösse und der inneren Struktur von Kolloidteilchen mittels Röntgenstrahlen. Nachr. Königl. Ges. Wiss. Göttingen..

[35] Sharma P., Rocha R.A., de Medeiros S.N., Paesano A., Hallouche B. (2007). Structural, Mössbauer and magnetic studies on Mn-substituted barium hexaferrites prepared by high energy ball milling. Hyperfine Interact..

[36] Townes W.D., Fang J.H., Perrota A.J. (1967). The crystal structure and refinement of ferrimagnetic barium ferrite, BaFe_12_0_19_. Z. Kristallogr..

[37] Routil R.J., Barham D. (1974). Occurrence of Strontium-Iron Oxide SrFe_12_O_19_ in the Fe_2_O_3_-Na_2_O-SrSO_4_ System. Can. J. Chem..

[38] Geiler A.L., Yoon S.D., Chen Y., Chinnasamy C.N., Chen Z., Geiler M., Harris V.G., Vittoria C. (2007). BaFe_12_O_19_ thin films grown at the atomic scale from BaFe_2_O_4_ and α-Fe_2_O_3_ targets. Appl. Phys. Lett..

[39] Moore P.B., Gupta P.K.S., Page Y.L. (1989). Crystal Structure of Magnetoplumbite. Am. Mineral..

[40] Ashima, Sanghi S., Agarwal A., Reetu (2012). Rietveld refinement, electrical properties and magnetic characteristics of Ca-Sr substituted barium hexaferrites. J. Alloys Compd..

[41] Shepherd P., Mallick K.K., Green R.J. (2006). Magnetic and structural properties of M-type barium hexaferrite prepared by co-precipitation. J. Magn. Magn. Mater..

[42] Vinnik D.A., Tarasova A.U., Zherebtsov D.A., Gudkova S.A., Galimov D.M., Zhivulin V.E., Trofimov E.A., Nemrava S., Perov N.S., Isaenko L.I. (2017). Magnetic and Structural Properties of Barium Hexaferrite BaFe_12_O_19_ from Various Growth Techniques. Materials.

[43] Wong-Ng W., McMurdie H., Paretzkin B., Hubbard C., Dragoo A. (1988). Standard X-Ray Diffraction Powder Patterns of Fourteen Ceramic Phases. Powder Diffr..

[44] Murashko M.N., Chukanov N.V., Mukhanova A., Vapnik E., Britvin S.N., Polekhovsky Y.S., Ivakin Y.D. (2011). Barioferrite BaFe_12_O_19_: A New Mineral Species of the Magnetoplumbite Group from the Haturim Formation in Israel. Geol. Ore Depos..

[45] Kraus W., Nolze G. (1996). POWDER CELL–A program for the representation and manipulation of crystal structures and calculation of the resulting X-ray powder patterns. J. Appl. Crystallogr..

[46] Gómez M.I., Lucotti G., de Morán J.A., Aymonino P.J., Pagola S., Stephens P.W., Carbonio R.E. (2001). Ab initio structure solution of BaFeO_2.8–*δ*_, a new polytype in the system BaFeO*_y_* (2.5 ≤ *y* ≤ 3.0) prepared from the oxidative thermal decomposition of BaFe((CN)_5_ NO)·3(H_2_O). J. Solid. State Chem..

[47] Gil de Muro I., Insausti M., Lezama L., Rojo T. (2005). Effect of the synthesis conditions on the magnetic and electrical properties of the BaFeO_3–*x*_ oxide: A metamagnetic behavior. J. Solid. State Chem..

[48] Winkler R., Ciria M., Ahmad M., Plank H., Marcuello C. (2023). A Review of the Current State of Magnetic Force Microscopy to Unravel the Magnetic Properties of Nanomaterials Applied in Biological Systems and Future Directions for Quantum Technologies. Nanomaterials.

[49] Gandhi A.C., Manjunatha K., Chan T.-S., Wu S.Y. (2022). Structural and Superconducting Proximity Effect of SnPb Bimetallic Nanoaloys. Nanomaterials.

[50] Krichevtsov B.B., Pavlov V.V., Pisarev R.V., Selitsky A.G. (1994). Linear magnetoelectric effect in magnetic garnet thin films. Ferroelectrics.

