# Supplementary material for: Thin Films of BaM Hexaferrite with an Inclined Orientation of the Easy Magnetization Axis: Crystal Structure and Magnetic Properties
Source: Nanomaterials (Basel). 2024 Nov 23;14(23):1883. doi: 10.3390/nano14231883 (PMC11643594; doi:10.3390/nano14231883)
Supplement: Supplementary file 1 [file nanomaterials-14-01883-s001.zip › nanomaterials-3264990-supplementary.pdf]

nanomaterials

Supplementary Materials

Thin Films of BaM Hexaferrite with an Inclined Orientation of the Easy Magnetization Axis: Crystal Structure and Magnetic Properties

Boris Krichevtsov [1,*], Alexander Korovin [1], Vladimir Fedorov [2], Sergey Suturin [1], Aleksandr A. Levin [1], Andrey Telegin [3], Elena Balashova [1] and Nikolai Sokolov [1]

[1] Ioffe Institute, Politechnicheskaya 26, 194021 St. Petersburg, Russia; amkorovin@mail.ioffe.ru (A.K.); suturin@mail.ioffe.ru (S.S.); aleksandr.a.levin@mail.ioffe.ru (A.A.L.); balashova@mail.ioffe.ru (E.B.); nsokolov@fl.ioffe.ru (N.S.)
[2] Laboratory of Renewable Energy Sources, Alferov University, Khlopin St. 8/3, 194021 St. Petersburg, Russia; burunduk.uk@gmail.com
[3] M.N. Mikheev Institute of Metal Physics, 18 S. Kovalevskaya Str., 620108 Yekaterinburg, Russia; telegin@imp.uran.ru
* Correspondence: boris@mail.ioffe.ru

Section S1. Optical Photos of Samples

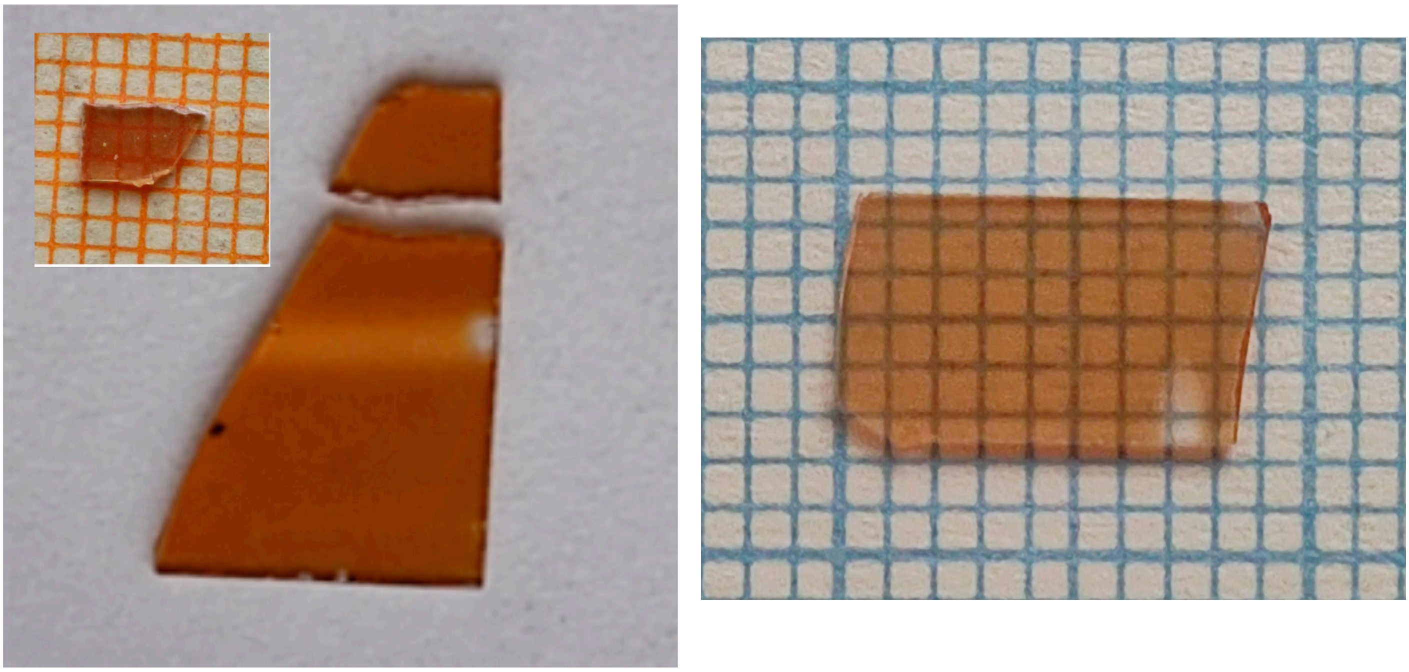

(a) (b)

Figure S1. Optical image of structure (**a**) #1 (thickness h = 50 nm) and (**b**) #2 (h = 50 nm).

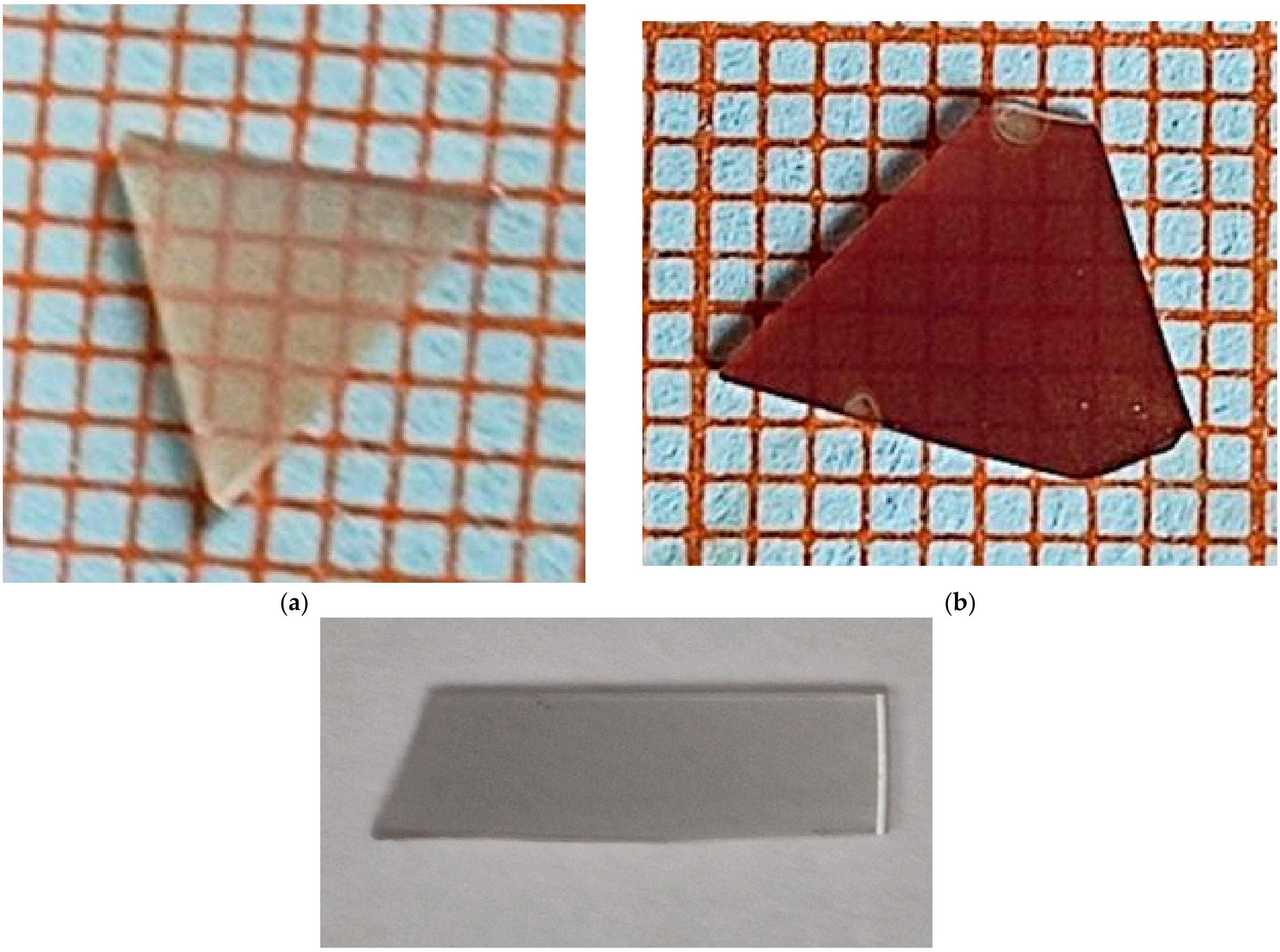

(**a**) (**b**) (**c**)

Figure S2. Optical image of structure (a) #3 (thickness h = 20 nm, (b) #4 (h = 170 nm), and (c) structure #7 (h = 50 nm) annealed in nitrogen atmosphere at T_{ann} = 950 °C.

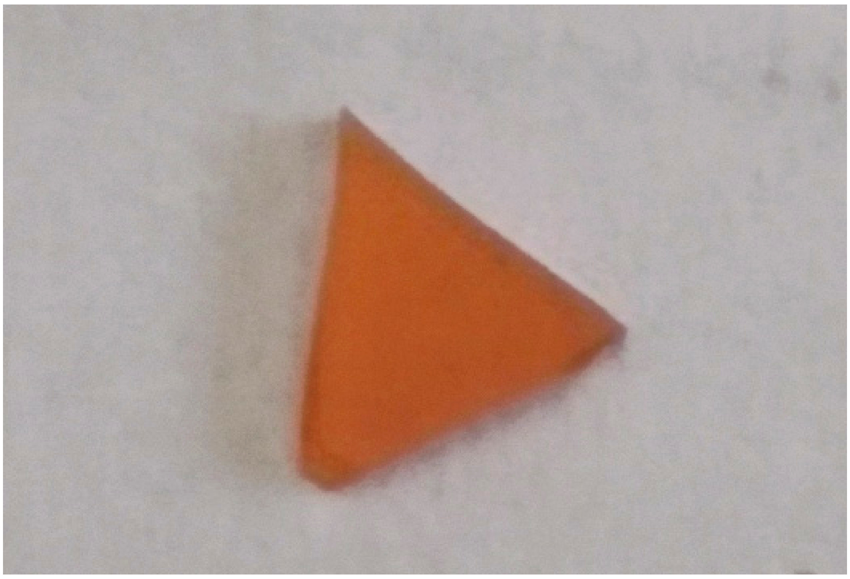

Figure S3. Optical image of structure #5 (thickness h = 50 nm).

Section S2. Powder XRD

Table S1. Unit cell parameters (*a*, *c*), unit cell volume (*V*), and interplane distances $d_{11–24}$ and $d_{22–48}$ corresponding, respectively, to reflections 11–24 and 22–48 of BaM and $BaFeO_{3–δ}$ phases according to current work and literature data.

a, Å<br>*c*, Å	*V*, Å^3	$d_{11–24}$, Å / $d_{22–48}$, Å	Sample	PDF-2 card	Ref.
$BaFe_{12}O_{19}$, space group $P6_3/mmc$ (194)					
– [a]<br>– [a]	701.7(3.2)	2.6024(3) / 1.3012(1)	50 nm film	–	this work
5.89<br>23.218	697.57	2.6263 / 1.3132 [b]	powder	01-075-9113	[1]
5.890(5) 23.218(3)	697.57(60)	2.6203 / 1.3102 [b]	powder	–	[2]
5.893<br>23.194	697.56	2.6268 / 1.3134 [b]	single crystal	–	[3, 4]
–<br>23.17(2)	–	– / –	650 nm film	–	[5]
5.892<br>23.198	697.44	2.6300 / 1.3130	–	00-027-1029	–
5.873(2) 23.007(6)	687.24(29)	2.6154 / 1.3077 [b]	single crystal	–	[6]
5.90<br>23.23	699.77	2.6301 / 1.3151	powder	01-080-6191	[7]
5.895<br>23.199	698.18	2.6276 / 1.3138 [b]	powder	–	[8]
5.8922(1) 23.1953(6)	697.40(2)	2.6266 / 1.3133 [b]	powder	–	[9]
5.8929(4) 23.194(2)	697.54(6)	2.6268 / 1.3134 [b]	powder	–	[9]
5.8915(2) 23.1917(8)	697.13(4)	2.6263 / 1.3131 [b]	powder	–	[9]
5.8962(4) 23.1927(1)	698.28(6)	2.6279 / 1.3140 [b]	powder	–	[9]
5.8948(3) 23.1780(8)	697.51(4)	2.6271 / 1.3135 [b]	powder	–	[9]
5.8917(9) 23.173(3)	696.60(19)	2.6259 / 1.3129 [b]	powder	–	[9]
5.8945(5) 23.215(3)	698.54(11)	2.6278 / 1.3139 [b]	powder	–	[10]
5.875(3) 23.137(19)	691.60(67)	2.6191 / 1.3096 [b]	mineral (powder)	–	[11]
$BaFeO_{2.654}$, space group $P6_3/mmc$ (194)					
5.77944(1)<br>24.60871(6)	711.854(2)	2.6207 / 1.3078<br>2.6207 / 1.3104 [c]	powder	01-070-7891	[12]
$BaFeO_{2.81}$[d], space group $P6_3/mmc$ (194)					
5.784(7)<br>24.63(3)	713.6(1.2)	2.6229 / 1.3088<br>2.6229 / 1.3115 [c]	powder	01-074-8665	[13]

[a] the cal[a] calculation of the parameters of the hexagonal cell is impossible, since there are only two reflections, and these are the first and second order of the same reflection with Miller-Bravais indices *hkil* = 11–24

[b] the values of *d* were calculated by the PowderCell program, version 2.4 [14] using the unit cell parameters of the BaM hexaferrite

[c] interplanar distances for reflections 10–18 and 2 0 –2 16 of the $BaFeO_{3–δ}$ compound

[d] the composition is given according to the results of the refinement of the structure [12] by the Rietveld method, while according to the PDF-2 card 01-070-7891, the composition is $BaFeO_{2.67}$

Section S3. Calculations in Stoner-Wolfarth model.

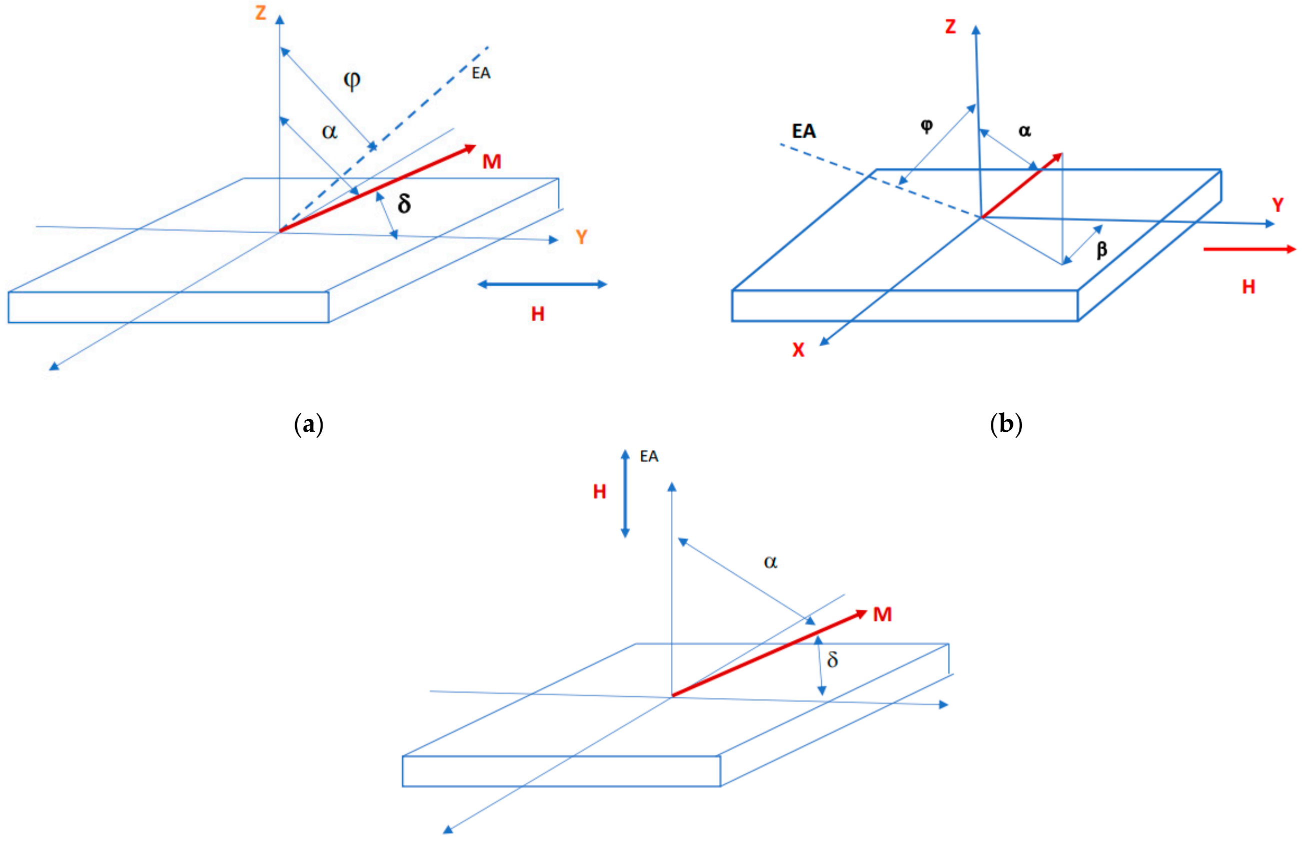

(**a**) (**b**)

(**c**)

Figure S4. Schematical drawing of coordinate system, orientation of Easy Axis of magnetization (EA), magnetization M, and in-plane magnetic field H for (a) $\theta = 0°$, (b) $\theta = 90°$, and (c) out-of-plane orientation of magnetic field. .

The calculation of the orientation of the magnetization M of the obtained films was carried out by minimizing the density of the thermodynamic potential W, which in the general case includes terms describing the interaction of the magnetization M with the external magnetic field H (Zeeman term), the energy of uniaxial magnetic anisotropy $E_a = K_u(u{\cdot}M)^2$, and the energy of demagnetizing fields $2\pi M_z^2$:

$$W = -(\mathbf{M}{\cdot}\mathbf{H}) - K_u(\mathbf{u}{\cdot}\mathbf{m})^2 + 2\pi M_z^2, \quad \text{(S1)},$$

where $K_u > 0$ is the uniaxial anisotropy parameter, **u** is the unit vector in the EA direction, and **m** is the unit vector in the **M** direction.

In the case of in-plane orientation of the magnetic field along Y axis and orientation of EA in ZY - plane (Figure S4a), the orientation of the magnetization is determined by the minimum of the expression:

$$W/M_s = -H{\cdot}\sin(\alpha) + (H_a/2){\cdot}\sin^2(\alpha - \varphi) + 2\pi M_s{\cdot}\cos^2(\alpha), \quad \text{(S2)}$$

i.e., as a solution of the equation:

$$\partial(W/M_s)/\partial\alpha = -H{\cdot}\cos(\alpha) + (H_a/2){\cdot}\sin(2(\alpha - \varphi)) - 2\pi M_s{\cdot}\sin(2\alpha) = 0, \quad \text{(S3)}$$

where M_s is the saturation magnetization, $H_a = 2K_u/M_s$. The calculations were performed for the values $4\pi M_s$ = 4.6 kG, the uniaxial anisotropy field H_a = 18 kOe, and the angle

between the EA and the normal to the surface $\theta = 0°$ (Figure S4a). Results of the calculations are presented in Figure S.5.

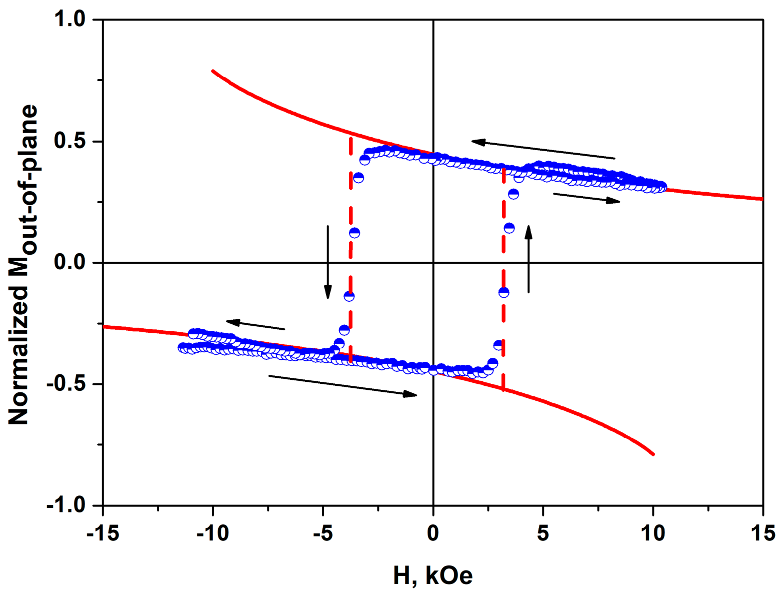

Figure S5. Experimental (blue symbols) and calculated (red dashed and solid lines) $M_{out-of-plane}(H_{in-plane})$ dependence for $\theta = 0°$.

For orientation of EA in the ZX plane perpendicular to the magnetic field **H** ($\theta = 90°$) (Figure S4b), the orientation of the magnetization **M** (i.e. angles α and β) can be calculated by solving the system of equations:

$$-H\cdot\cos(\alpha)\cdot\cos(\beta) - (H_a/2)\cdot[\ \sin(2\alpha)\cdot(\ \sin(2\varphi)\cdot\sin(2\beta) - \cos(2\varphi) + \cos(2\alpha)\cdot\sin(2\varphi)\cdot\sin(\beta)] - 2\pi M_s\cdot\sin(2\alpha) = 0 \qquad \text{(S4)}$$

$$H\cdot\sin(\alpha)\cdot\sin(\beta) - H_a\cdot(\cos(\varphi)\cdot\cos(\alpha) + \sin(\varphi)\cdot\sin(\alpha)\cdot\sin(\beta))\cdot\sin(\alpha)\cdot\sin(\varphi)\cdot\cos(\beta) \ = 0 \qquad \text{(S5)}$$

Results of calculations for $4\pi M_s = 4.6$ kG, $H_a = 18$ kOe, and $\phi = 58°$ are presented in Figure S6. Magnetic field dependences of m_x, m_y, and m_z (components of unit vector **m** along axes X, Y, and Z, respectively) do not show any hysteresis behavior and are related to reciprocal magnetization rotation. In vicinity of $H \cong \pm17$ kOe, i.e., close in absolute magnitude to $H_a = 18$ kOe, both the $m_z(H)$ and $m_y(H)$ dependences demonstrate behavior typical to second-order phase transitions, and $m_x(H)$ dependence reveals the change of slope. In magnetic field region -10 kOe $< H <$ 10 kOe, the change in m_z-component is rather small in accordance with hysteresis loop shown in Figure 6a of the manuscript (panel 87°). Some hysteretic behavior observed in the panel may be attributed to not exact orientation of magnetic field **H** along Y axis or to small inhomogeneous in orientation of EA in the sample.

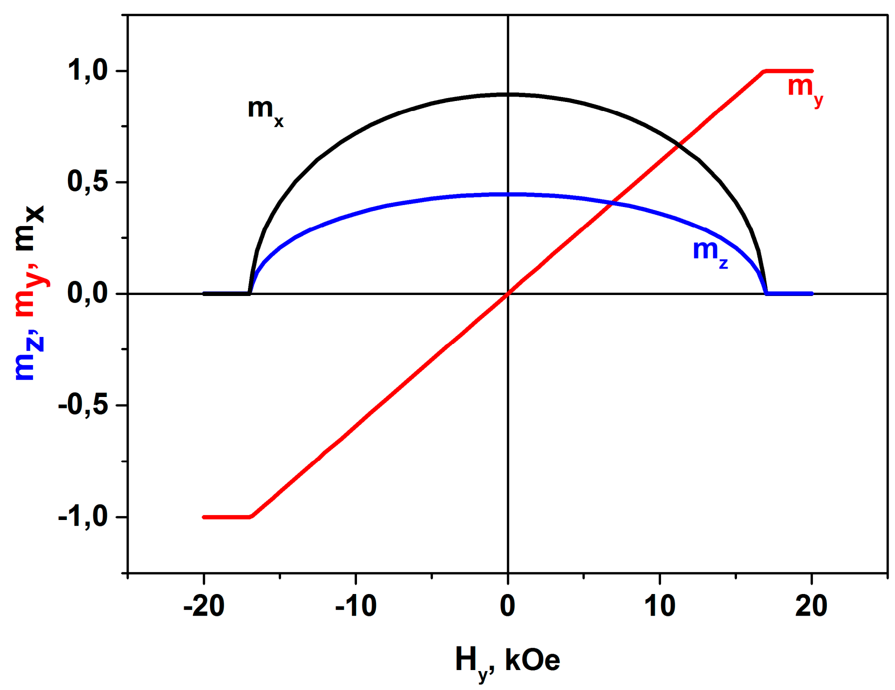

Figure S6. Magnetic field dependences of m_x, m_y, and m_z for orientation of in-plane magnetic field $\mathbf{H}_{\text{in-plane}}$ along *Y*-axis and orientation of EA in XZ-plane (θ = 90°).

In the case of out-of-plane orientation of the magnetic field (Figure S4c), the orientation of the magnetization is determined by the solution of the equation:

$$\mathrm{H}\cdot\sin(\alpha) - (H_a/2)\cdot\sin2(\alpha-\varphi) - 2\pi M\cdot\sin(2\alpha) = 0 \quad \text{(S6)}$$

The results of calculations are shown in Figure S7.

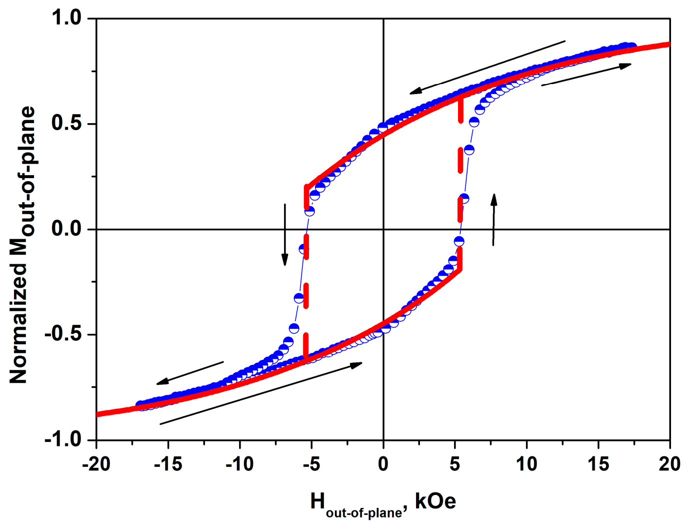

Figure S7. Experimental (blue symbols, blue thin solid line is a guide for eye) and calculated (red thick dashed and solid lines) dependence $M_{\text{out-of-plane}}(H_{\text{out-of-plane}})$.

The calculations in the Stoner-Wolforth model are valid in the case of uniform magnetization, when there are no magnetic domains and domain walls. They describe reversible rotations of magnetization caused by the applied magnetic field. Magnetization jumps

associated with the creation and movement of domain walls in Figures S5 and S7 are shown by vertical lines.

References

1. Sharma, P.; Rocha, R.A.; de Medeiros, S.N.; Paesano jr, A.; Hallouche, B. Structural, Mössbauer and magnetic studies on Mn-substituted barium hexaferrites prepared by high energy ball milling. *Hyperfine Interact* **2007**, *175*, 77 – 84. https://doi.org/10.1007/s10751-008-9591-2.
2. Sharma, P.; Rocha, R.A.; Medeiros, S.N.; Hallouche, B.; Paesano jr, A. Structural and magnetic studies on mechanosynthesized $BaFe_{12-x}Mn_xO_{19}$. *J. Magn. Magn. Mater.* **2007**, *316*, 29 – 33. https://doi.org/10.1016/j.jmmm.2007.04.007
3. Townes, W.D.; Fang, J.H.; Perrota, A.J. The crystal structure and refinement of ferrimagnetic barium ferrite, $BaFe_{12}0_{19}$. *Z. Kristallogr.* **1967**, *125*, 437 – 449. https://doi.org/10.1524/zkri.1967.125.125.437
4. Routil, R.J.; Barham, D. Occurrence of Strontium-Iron Oxide $SrFe_{12}O_{19}$ in the Fe_2O_3-Na_2O-$SrSO_4$ System. *Can. J. Chem.* **1974**, *52*, 3235 – 3246.
5. Geiler, A.L.; Yoon, S.D.; Chen, Y.; Chinnasamy, C.N.; Chen, Z.; Geiler, M.; Harris, V.G.; Vittoria, C. $BaFe_{12}O_{19}$ thin films grown at the atomic scale from $BaFe_2O_4$ and α-Fe_2O_3 targets. *Appl. Phys. Lett.* **2007**, *91*, 162510. https://doi.org/10.1063/1.2800890
6. Moore, P.B.; Gupta, P.K.S.; Page, Y.L. Crystal Structure of Magnetoplumbite. *Am. Mineral.* **1989**, *74*, 1186–1194.
7. Ashima; Sanghi, S.; Agarwal, A.; Reetu. Rietveld refinement, electrical properties and magnetic characteristics of Ca-Sr substituted barium hexaferrites, *J. Alloys Compds.* **2012**, *513*, 436. https://doi.org/10.1016/j.jallcom.2011.10.071
8. Shepherd, P.; Mallick, K.K.; Green, R.J. Magnetic and structural properties of M-type barium hexaferrite prepared by co-precipitation. *J. Magn. Magn. Mater.* **2006**, *311*, 683 – 692. https://doi.org/10.1016/j.jmmm.2006.08.046
9. Vinnik, D.A.; Tarasova, A.Yu., Zherebtsov, D.A.; Gudkova, S.A.; Galimov, D.M.; Zhivulin, V.E.; Trofimov, E.A.; Nemrava,S.; Perov, N.S.; Isaenko, L.I.; Niewa, R. Magnetic and Structural Properties of Barium Hexaferrite $BaFe_{12}O_{19}$ from Various Growth Techniques. Materials **2017**, *10*, 578. https://doi.org/10.3390/ma10060578
10. Wong-Ng, W.; McMurdie, H.; Paretzkin, B.; Hubbard, C.; Dragoo, A. Standard X-Ray Diffraction Powder Patterns of Fourteen Ceramic Phases. *Powder Diffr.* **1988**, *3*, 249 – 254.. https://doi.org/10.1017/S0885715600013579
11. Murashko, M.N.; Chukanov, N.V.; Mukhanova, A.; Vapnik, E.; Britvin, S.N. Polekhovsky, Yu.S.; Ivakin Yu.D. Barioferrite $BaFe_{12}O_{19}$: A New Mineral Species of the Magnetoplumbite Group from the Haturim Formation in Israel. *Geol. Ore Depos.* **2011**, *53*, 558 – 563. https://doi.org/10.1134/S1075701511070142
12. Gómez, M.I.; Lucotti, G.; de Morán, J.A.; Aymonino, P.J.; Pagola, S.; Stephens, P.W.; Carbonio, R.E. Ab initio structure solution of $BaFeO_{2.8-\delta}$, a new polytype in the system $BaFeO_y$ ($2.5 \leq y \leq 3.0$) prepared from the oxidative thermal decomposition of $BaFe((CN)_5\ NO)\cdot 3(H_2O)$. *J. Solid State Chem.* **2001**, *160*, 17– 24. https://doi.org/10.1006/jssc.2001.9119
13. Gil de Muro, I.; Insausti, M.; Lezama, L.; Rojo, T. Effect of the synthesis conditions on the magnetic and electrical properties of the $BaFeO_{3-x}$ oxide: a metamagnetic behavior. *J. Solid State Chem.* **2005**, *178*, 1712–1719. https://doi.org/10.1016/j.jssc.2005.03.028
14. Kraus, W.; Nolze, G. *POWDER CELL* - a program for the representation and manipulation of crystal structures and calculation of the resulting X-ray powder patterns', *J. Appl. Crystallogr.* **1996**, *29* (1996) 301–303. doi:10.1107/S0021889895014920.

Disclaimer/Publisher's Note: The statements, opinions and data contained in all publications are solely those of the individual author(s) and contributor(s) and not of MDPI and/or the editor(s). MDPI and/or the editor(s) disclaim responsibility for any injury to people or property resulting from any ideas, methods, instructions or products referred to in the content.
